# A Distributed Computing Solution Based on Distributed Kalman Filter for Leak Detection in WSN-Based Water Pipeline Monitoring

**DOI:** 10.3390/s20185204

**Published:** 2020-09-12

**Authors:** Valery Nkemeni, Fabien Mieyeville, Pierre Tsafack

**Affiliations:** 1 Université Claude Bernard Lyon 1, Ampère-CNRS UMR5005, F-69621 Villeurbanne, France; fabien.mieyeville@univ-lyon1.fr; 2 University of Buea, Faculty of Engineering and Technology, P.O. Box 63 Buea, Cameroon; tsafack.pierre@ubuea.cm

**Keywords:** distributed computing, wireless sensor networks, distributed Kalman filter, water pipeline monitoring, distributed data fusion

## Abstract

Wireless Sensor Network (WSN) applications that favor more local computations and less communication can contribute to solving the problem of high power consumption and performance issues plaguing most centralized WSN applications. In this study, we present a fully distributed solution, where leaks are detected in a water distribution network via only local collaborations between a sensor node and its close neighbors, without the need for long-distance transmissions via several hops to a centralized fusion center. A complete approach that includes the design, simulation, and physical measurements, showing how distributed computing implemented via a distributed Kalman filter improves the accuracy of leak detection and the power consumption is presented. The results from the physical implementation show that distributed data fusion increases the accuracy of leak detection while preserving WSN lifetime.

## 1. Introduction

A Wireless Sensor Network (WSN) consists of several embedded nodes with sensing, processing and wireless communications capabilities, distributed over an area of interest to monitor physical or environmental conditions [1]. They are spatially distributed systems that exploit wireless communication as the means of communication between nodes and are typically constrained in terms of energy, computing power, memory and communication bandwidth due to their requirements of a small size and low power consumption [2]. Application areas of WSNs include geographical monitoring, habitat monitoring, transportation, military systems, business processes, microclimate research, medical care and others [3,4]. In this study, we look at the state of the art of distributed computing in WSN and propose a distributed solution to improve the accuracy of leak detection in a WSN-based water pipeline monitoring system using low-cost vibration sensors.

### 1.1. WSN: Shifting towards a Distributed Approach

Most WSN monitoring applications in the literature are centralized [4,5]. This leads to the underutilization of the processing unit and overutilization of the communication unit of sensor nodes since the primarily role of the sensor nodes in such centralized architectures is to collect and transmit data periodically to an intelligent central base station where all the processing is done in order to detect anomalous behaviors [4,6,7]. In addition, in large-scale monitoring applications, most of the sensor nodes are geographically far away from the base station and are usually battery powered [8]. Therefore, the drawbacks of WSNs with centralized architectures deployed in large-scale monitoring applications are enormous bandwidth requirement and huge energy consumption as periodic transmission of raw data over long distances, via multiple hops to the base station leads to fast depletion of sensor node’s battery and shortens the lifespan of a WSN [7,9,10]. Other drawbacks include low reliability, longer response time, high bandwidth cost, low level data security and privacy [7,9,10,11,12]. Some works in the literature have demonstrated the feasibility of distributed computing in WSN and its promises of gain in performance and lower power consumption [5,7,11,12,13]. By performing more local computation, limiting exchanges only between neighboring nodes and reducing the number of messages that need to be transmitted, distributed computing in WSN has the potential of providing a solution to the drawbacks of the centralized approach [12,13,14,15].

### 1.2. The Stakes of Water Supply in Developing Countries

Water is a basic necessity for everyday life and for the effective accomplishment of many industrial processes. In most communities, water transportation via pipelines to users seems to be the most economical way [16] and consists of water supply systems comprising of two different parts: (1) Transmission mains, which are pipes responsible for transporting water to tanks and (2) Water Distribution Networks (WDN), which are pipes and service connections for distributing water to users. However, these infrastructures are not completely watertight as even in the most recent and well-built WDN, some level of leakage and occasional pipe bursts occur, leading to water losses [17].

Water pipeline leakages are one of a few challenges to the water utility companies all over the world as water loss through leakages is recognized as a costly problem worldwide, due to the waste of precious liquid, as well as from the economic point of view [18,19,20]. A report published by the World Bank in 2016 indicated that in developing countries, roughly 45 million cubic meters of water are lost daily with an economic value of over US $3 billion per year. The report also stated that saving half of those losses would provide enough water to serve at least 90 million people [21]. In Cameroon, the level of Non-Revenue Water (NRW), which is the portion of the total amount of water produced for which the water utility company generates no income from (because it is lost via leaks/burst and/or theft) is at 4.67% [22,23]. The reason for this level of NRW is explained by the aging infrastructure of the WDN that creates physical losses through leaks and/or bursts.

Water demand is increasing continuously and rapidly as a result of the growth of the Earth’s population, but water resources are facing a problematical and constant decrease caused by global heating and climate change [18,24]. Unlike other more peculiar phenomena, water scarcity is common to both developing and developed countries [18]. The scarcity of water thus requires that water losses due to leaks be minimized by accurately detecting and localizing leakages in real time, each time they occur. This has led to enormous research over the years in the field [25,26,27,28,29,30,31,32], providing a wide range of methods for detecting and locating leaks in water pipelines.

### 1.3. Problem Statement

In recent years, WSN-based Water Pipeline Monitoring (WWPM) using vibration sensors has become popular since the sensors offer the distinct advantage of providing real-time monitoring of water distribution pipelines, which can prompt immediate interventions [33]. Vibration-based WSNs can be used for pipeline monitoring because water pipeline pressure monitoring can be transformed into acceleration monitoring of the pipe surface since from studies such as [25,34,35], a transient change in pressure is always accompanied by an increase in the pipe surface acceleration at the corresponding locations along the pipe length [34]. In addition, vibration sensors (accelerometers, piezoelectric transducer, force sensitive resistors, etc.) are easy to install and less costly to maintain/operate. While previous vibration-based WSNs have been useful in detecting leakages, they still have the challenge of reliably detecting leaks in the midst of random environmental noise since they make use of low-cost sensors with low accuracy [33,35,36]. However, since WWPM makes use of multiple low-cost sensors to monitor the pipeline, multi-sensor data fusion techniques, which have been successfully used in target tracking [37], can be used to increase the reliability of leak detection systems based on low-cost vibration sensors [36]. Multi-sensor data fusion combines redundant data from multiple low-cost sensors to achieve a more accurate information whose quality exceeds that achieved by using a single sensor [36,38]. In addition, the low-power consumption requirement and the need for a WWPM to go unattended for a long period of time without any replacement of the sensor node’s battery [33,39,40], affects the choice of multi-sensor data fusion technique that can be used. Multi-sensor data fusion in WSN can either be done via a centralized, decentralized or in a distributed manner [37,41]. The centralized data fusion technique will require multi-hop communications, which have a higher probability of developing an energy hole in the network, thus shortening the lifespan of the WSN [12,14,15]. Thus, using distributed data fusion may increase the lifespan of the WSN as there is no central point for fusion and multi-hop communications will be eliminated entirely. The objective of distributed data fusion is to use distributed computations across the network such that the local information at each sensor node converges to the optimal value of the centralized fusion approach [42].

### 1.4. Objectives of the Study

The purpose of this research is to demonstrate the benefits of trading off long-distance multi-hop communications for computation in WSNs by exploiting the sensor node’s processing unit to implement distributed computing. In some recent studies, distributed computing in WSNs has been proven to be much more efficient in terms of energy and performance than the traditional centralized computation schemes used in most WSN monitoring applications. However, most of these studies are validated based on simulations [11,12,13]. Our work provides a complete systemic approach, involving simulations, physical system design, field deployment and experimental validation. The objectives of our study include: (1) investigate the feasibility of implementing a completely distributed solution for leak detection in WSN-based Water Pipeline Monitoring (WWPM), (2) determine the effect of distributed data fusion on the accuracy of leak detection and (3) measure the power consumption. We start by simulating the distributed solution and its power consumption, we design the wireless sensor node, then move to the field deployment which involves detecting leaks in water pipes using the proposed distributed approach and also measuring the power consumption. In this study, we implement distributed computing by using a distributed Kalman filter algorithm and then applied it to leak detection in WWPM. The results indicate an increase in the accuracy of leak detection when a distributed Kalman filter is used. In addition, we measure the power consumption of our solution.

### 1.5. Organization of the Paper

The rest of the paper is structured as follows: Section 2 provides a brief review of distributed computing in WSN, its advantages and a brief survey of some studies implementing distributed computing in WSN. In Section 3, we present the state of the art of water pipeline monitoring by classifying leak detection techniques and reviewing some related works in WSN-based Water Pipeline Monitoring (WWPM) using nonintrusive sensors. A detailed description of our proposed node architecture, and the distributed Kalman filter implemented, is presented in Section 4. In Section 5, we describe the simulation setup, the physical testbed used to demonstrate our solution and the power consumption measurement device, while Section 6 presents the results obtained from simulations and field experiments alongside with the discussions and, finally, Section 7 concludes the paper.

## 2. Distributed Computing in Wireless Sensor Networks

In this section, we provide a brief review of distributed computing in WSNs, looking at the motivation for distributed computing in WSNs, the benefits of distributed computing in WSNs, and presenting a brief survey of some studies that have applied distributed computing in WSNs.

### 2.1. The Relevance of Distributed Computing in WSN

Despite their distributed architecture, most WSN monitoring applications are centralized, where most of the intelligence is found out of the WSN and processing takes place at a central base station. Sometimes, data collected from the WSN and present at the base station are transmitted to the cloud by the base station for further processing [12]. The sensor nodes serve only as data collectors with the sole responsibility of sensing certain phenomena of the physical world and transmitting it to the central node for processing. In such cases, there is little or no intelligence at the sensor node level and the sensor nodes act primarily in a sense-only fashion [43]. Most sensor nodes in large-scale monitoring applications are far from the base station and will require enormous energy to transmit their data via a single hop to the base station. This leads to the transmission of sensor node’s data to the base station via multi-hop communications, since it is well known that the energy required for two sensor nodes to communicate decreases as the distance between them decreases, often according to an inverse square law. The multi-hop communications lead to an increase in the number of data transmission and thus increases the overall power consumption and shortens the lifespan of a WSN by decreasing the lifetime of every node serving as a relay. Furthermore, in the centralized approach, nodes directly connected to the base station will be involved in relaying all the messages directed to the base station. This results in what is referred to as the energy hole effect in some studies [12,14,15]. The energy hole effect is caused by the uneven distribution of the energy consumption among the nodes in the network. The energy of nodes directly connected to the base station depletes faster since they are involved in relaying all the messages directed to the base station. As the energy of these nodes get expended, the remaining one-hop neighbors of the base station will face an even greater load, thus creating an avalanche effect that can quickly disable the network [12].

With all the drawbacks of the centralized computing scheme in WSN, a logical thing to do is to perform distributed computing within the WSN. The core idea is to invest more into computation within the network by harnessing the onboard processing capabilities embedded in each node for local processing, alongside with local communication, i.e., those that occur between neighboring sensor nodes in a WSN, whenever possible to save on communication costs. By distributed computing, each node performs processing on its local data and only communicates with its direct neighbors to reach some desired accuracy, thus, minimizing the amount of multi-hop communications as much as possible through local collaboration among sensor nodes.

As a result of the fast development in microelectronics, more powerful sensor node processing units (microcontrollers) are being developed with higher computational and memory capability but consuming lower power [2,44,45,46]. This is reducing the challenges of embedding intelligent data processing on sensor nodes. For this reason, distributed computing is becoming increasingly popular in most WSN monitoring applications [2,44,45]. The presence of distributed sensing in a WSN and the availability of computing resources at each sensing node can be properly harnessed by using distributed algorithms that minimize communication and energy costs as well as provide robustness to node failures [11]. This creates a scenario where the sensor nodes can communicate among themselves and perform distributed computation over the sensed data to identify the occurrence of an event [4]. This improves on the scalability of WSNs, reduces latency as well as network energy consumption, and also improves data security and privacy [8,10,12].

### 2.2. WSN and Edge Computing

In the last decade, the term Internet of Things (IoT), has progressively gained dominance as the keyword to define connected embedded devices. It replaces the pioneer term WSN, which is one of the first physical implementations of Ubiquitous Computing, and finally integrates it as a part of IoT (the physical network mainly used for monitoring).

With research progress in this field, numerous computing paradigms have emerged such as Mobile Cloud Computing (MCC), cloudlet computing, mobile clouds, mobile IoT computing, IoT cloud computing, fog computing, Mobile Edge Computing (MEC), and edge computing [47]. The latter, edge computing [48], could be considered as a related field of WSN distributed computing. However, what differs between WSN and IoT is the hardware computation capacity. Recently, the Internet Engineering Task Force (IETF) [49] standardized a classification for devices that use the Internet that demonstrates that a performance gap exists between the lowest class, i.e., Class 0, dedicated to WSN [50] and Class 1 and above, which comprise the hardware commonly found in IoT.

Furthermore, given that wireless sensor nodes are highly constrained in terms of computation capacity and energy consumption, the promising capacity of edge computing has not been evaluated here due to the gap between the hardware capacity of connected devices involved in each of the networks (WSN and IoT) [51] and those developed using a state-of-the-art energy-aware hardware design for edge computing by Jiang et al. [52]. Even though paradigms and algorithmic propositions emerging from edge computing could be of interest, issues such as portability efforts and shrinking requirements needed to transfer software from medium- to high-performance computing units to highly energy-constrained and low-power computation capacity hardware targets led the authors to focus this study on distributed computing for low-cost/low-performance, battery-powered and highly constrained sensor networks [37].

### 2.3. A Survey on Distributed Computing in WSN

According to Stula et al. [53], distributed computation is any process conducted by multiple agents or entities that perform operations on information and together generate resulting information, and can be defined and observed in terms of memory, communication, and processing. Huang, in [5], classified the applications of distributed computing in WSN according to the following taxonomy: Distributed Query Processing, Collaborative Signal Processing, or Distributed Estimation and Detection. Other applications of distributed computing in WSN include: local (in-node or on-sensor or edge) processing and in-network processing.

In-network processing involves the processing of data as it travels via the WSN to the sink. For example, Serpen and Liu [13] demonstrated through simulations, a case study that leverages existing WSNs as a parallel and distributed hardware platform to implement computations for artificial neural network algorithms. The results of their simulation suggest that the WSN-based neurocomputing architecture is a feasible alternative for realizing parallel and distributed computation of artificial neural network algorithms. However, their study does not consider the energy constraint of WSN as they assumed that the sensor nodes are not limited in energy supply. In another study, Pascale et al. [12] proposed an in-network processing framework to tap into the collective computation capability of Internet of Things (IoT) devices by coupling data communication and processing for the transformation of raw data into appropriate actions as it travels via the network towards the actuating nodes. Their results showed that distributed computing decreases the latency and improves the distribution of energy consumption among the sensor nodes, thus mitigating the energy hole effect and increasing the expected lifetime of the network. However, the results were validated by simulations in Cooja.

In collaborative signal processing, also referred to as Wireless Distributed Computing (WDC), a master node which needs to perform a complex computational task in a limited time frame divides the computational task into a number of subtasks and then assigns these subtasks to some slave nodes (neighboring nodes) [54]. In [9], the authors discussed some of the possible applications of WDC such as image processing and pattern recognition, distributed data storage and database search, Synthetic Aperture Radar (SAR) processing, etc. Energy savings in WDC were demonstrated in this study by a wireless ad-hoc network comprised of a tactical handheld, radio nodes attached to a UAV, or sensor nodes. The findings showed that the reduction in energy consumption of the wireless nodes is achieved firstly by the fact that WDC enables processing within the network which reduces number of bits transmitted over the long backhaul at the cost of computational energy consumption. Chiasserini in [55] extended the concept of collaborative signal processing in WSN by using a collaborative computational algorithm and communication scheme where the sensor nodes were made to operate as a Distributed Digital Signal Processor (DDSP). Fast Fourier Transform (FFT) algorithm was applied to the DDSP approach and the results showed that the energy consumption obtained at different processing frequencies when the FFT is computed by a single sensor node were higher compared to the results derived in the case where the computation is distributed among multiple sensor nodes.

In the case of local processing, raw data are processed locally at the sensor node using its processing unit, and only the analyzed results are transmitted via the WSN to the sink. Feng et al., in [56], implemented an envelope analysis algorithm on a WSN node composed of a cortex-M4F core processor for feature fault extraction in a condition monitoring application using vibration sensors. The results from the study showed that the sensor node was able to identify simulated faults and achieve real-time condition monitoring while reducing the data transmission throughput by 95%. In another study, Kartakis et al. [8], presented an end-to-end water leak localization system, which exploits edge processing in battery-powered sensor nodes. The sensor nodes were based on Intel Edison development boards and NEC Tokin ultra-high-sensitivity vibration sensors and the proposed system combined a lightweight edge anomaly detection algorithm based on a Kalman filter and compression rates and a localization algorithm based on graph theory. According to the authors, the edge anomaly detection and localization elements of the systems produce a timely and accurate localization result and reduce the communication by 99% compared to the traditional periodic communication.

In distributed state estimation and detection applications, the WSN makes a decision about the value of a physical variable (estimation) or the occurrence of an event (detection) in a distributed manner. Distributed State Estimation (DSE) algorithms implement distributed data fusion, where neighboring nodes communicate with each other to improve the accuracy of the monitored parameter. In [57], the performance of a local Kalman filter and a distributed Kalman filter is evaluated experimentally using an ultrasound-based positioning application composed of a sensor network with seven sensor nodes. There is no centralized computation and the goal is to make sure that every node in the network has an accurate estimate by performing computations in a distributed manner and communicating only once per sampling interval. Battistelli et al., in [58] presented a novel event-triggered distributed state estimator based on a consensus Kalman filtering approach as well as a transmission triggering condition, while He et al., in [37], reviewed distributed Kalman filter algorithms for low-cost sensor networks.

In this study, we experimentally demonstrate the ability of distributed computing to improve the reliability of anomaly detection in WSN monitoring since most of the works available in the literature are based on simulations. We compare local processing (implemented by a local Kalman filter) and distributed state estimation (implemented by a distributed Kalman filter) based on results obtained from simulations and physical experiments, in a WWPM system that uses low-cost vibration sensors for leak detection.

## 3. State of the Art of Water Pipeline Monitoring

In this section, we first perform a general classification of the leak detection technique used in water pipeline monitoring and then focus on WWPM. We review some works in the literature that are closely related to our study and which use WSNs with nonintrusive sensors for leak detection in WDN. The survey is based on the node architectures, the type of sensors and pipe material used, the location where the leak signals are analyzed to detect the presence or absence of leaks, and the leak detection algorithm implemented in each of these studies.

### 3.1. Classification of Leak Detection Techniques

**Figure 1:**
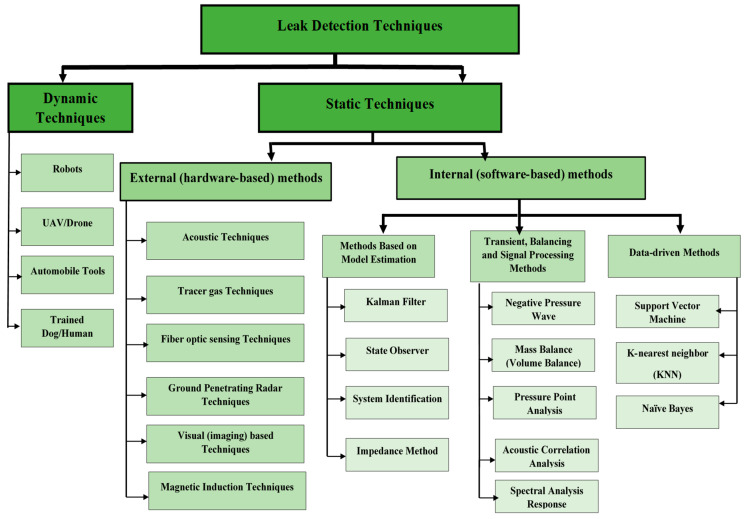
Classification of leak detection techniques.

The detection and localization of water pipeline leakages are important to water utility companies because of the need to conserve raw/treated water and save associated costs [19]. A lot of research efforts have been dedicated to the development of a vast variety of techniques for leak detection and localization to minimize water losses caused by leaks [30]. Based on their technical approach, Adedeji et al. [20], Baroudi et al. [36], Adegboye et al. [59], and Torres et al. [29] in their survey papers on pipeline monitoring categorized leak detection techniques in water pipelines as either external or internal. In other surveys, Ismail et al. [25] classified leak detection techniques into software-based methods and hardware-based methods while Chan et al. [60] classified them as active and passive systems. The software-based and hardware-based methods of Ismail et al. [25] and active and passive systems of Chan et al. [60] are likened to the internal and external methods of [20,29,36,54], respectively, as shown in Figure 1. In another study, El-Zahab et al. [30] classified leak detection systems into two major classes, i.e., static leak detection systems and dynamic leak detection systems. Static leak detection systems rely on sensors capable of sensing leak signals, coupled with a communication technology while dynamic leak detection systems require the mobilization of a leak inspection team that delivers the devices to the suspected leak site to perform an inspection and confirm or clear the suspicion [30]. Most software-based (internal or active) and hardware-based (external or passive) leak detection techniques are static, as shown in Figure 1.

In the sub-sections below, we briefly explain software-based and hardware-based methods of leak detection, highlighting the various techniques in each of the categories and stating their advantages and disadvantages.

#### 3.1.1. Software-Based Methods

The software-based methods use field sensors to monitor the operational and hydraulic conditions of the pipeline, such as the measurement of the flow, pressure and temperature [16,25,36,54] and smart computational algorithms to process the measurements in order to detect and localize the occurrence of leaks on the pipeline [29]. Some of the software-based methods available in the literature include methods based on model estimation (e.g., Kalman filter [19,29,61], state observer [62], system identification [63], the impedance method [64]), methods based on signal processing (Negative Wave Pressure [32], Mass Balance [65], Pressure Point Analysis [66], Acoustic Correlation Analysis [67], Spectral Analysis Response [68]) and data-driven methods (Support Vector Machine [69], K-nearest neighbor [70], Naïve Bayes [69]). They involve the use of either intrusive sensors or non- intrusive sensors to monitor the internal pipeline parameters. Those that make use of intrusive sensors are difficult to install but provide high accuracy whereas those that make use of nonintrusive sensors are easy to install but provide low accuracy in leak detection. As an advantage, they can make use of a WSN and they are also cost effective.

#### 3.1.2. Hardware-Based Methods

The hardware-based methods detect the presence of leaks from outside the pipeline by visual observation or by using specialized equipment that range from simple listening rods to more sophisticated approaches as inspection gauges sending magnetic fields, electromagnetic waves or ultrasound through a pipeline’s walls [16,25,36,54] for physical monitoring. They use local sensors to send an alarm when a leak occurs, and do not perform computation for diagnosing a leak [29]. Some examples available in the literature include acoustic techniques [71], tracer gas techniques, fiber optic sensing techniques [72], ground-penetrating radar techniques [73], magnetic induction techniques [74], etc. As a disadvantage, they may involve the use of expensive instruments, some are labor intensive and also do not make use of WSNs in monitoring. As an advantage, they are highly sensitive to leaks.

Figure 1 illustrates the classification of leak techniques into static and dynamic methods, followed by hardware-based and software-based methods. A detailed survey of these techniques along with a comparison can be found in [16,20,25,31,36,54,55]. In this study, our focus is on leak detection in WDN using WSN. The need to explore pipeline monitoring schemes that incorporate WSNs is advantageous because WSNs are easy to deploy and flexible enough to be installed in any environment. WSNs provide effective solutions for pipeline monitoring, due to its low cost, flexibility and ease of deployment in inaccessible terrain [16,33,36].

### 3.2. WSN-Based Water Pipeline Monitoring

#### 3.2.1. Introduction

WSN-based Water Pipeline Monitoring (WWPM) systems are practically developed from two main parts: the sensors/equipment installed along the pipeline that periodically collect useful information relating to some pipeline parameters and the algorithms that process this information in order to detect and localize leaks in the pipeline [26]. Typically, the remote field sensors provide data to a centralized monitoring station (for centralized systems) or fusion center (for decentralized systems), where the data undergo filtering, signal processing and are later fed into leak detection algorithms to identify a leak. The fusion center used in decentralized systems is a local aggregation unit present in the field and may have a direct communication with the base station, which collects data from the sensor nodes within a cluster and locally processes them [32,37,41,75]. In most leak detection systems, a difference between the measured and predicted operational parameters indicates a leak [36]. The biggest problem with leak detection in WWPM using low-cost sensors is that the leak signals may be inaccurate due to the low sensitivity of the sensors and environmental noise and may result in false alarms in the leak detection system. Thus, the issue of reliably identifying a leak signal in the midst of errors from a number of sources (commonly called noise) is a fundamental challenge of any leak detection system [33,76].

Depending on where the leak signals from remote sensors are filtered, where preprocessing of the leak signals takes place and where the leak detection and leak localization algorithms are executed, WWPM solutions can be classified as either centralized, decentralized or distributed. In order to have a good understanding of all the significant WWPM approaches available in the literature, we propose the criteria given in Table 1, which provides a description of the attributes we used to classify WWPM studies available in the literature. Our focus is on WWPM studies that monitor the pipe surface vibration as an indirect method of monitoring the pressure fluctuations caused by leaks in the pipeline and that make use of nonintrusive (vibration-based) sensors (e.g., accelerometers, piezoelectric transducers, force sensitive resistor, etc.). The algorithms for processing the leak data (pipe surface vibration) used in these studies fall in the transient, balancing and signal processing methods of leak detection techniques shown in Figure 1.

#### 3.2.2. A Review on WWPM Studies

A challenge associated with all vibration-based pipeline leak detection techniques is the possibility of potential false alarms caused by environmental perturbations unrelated to the state of the pipe [33,35,77]. In addition, the sensor needs to be placed at a small distance from the leak, since wave attenuation in plastic pipes is strong [68]. This will result in an increase in the number of sensors required for leak detection and will result in a higher system cost if high-cost sensors are used. The use of low-cost vibration sensors can reduce the cost of the leak detection system, but the leak detection system will be prone to false alarms since low-cost vibration sensors have a lower accuracy. In this sub-section, we focus on some representative vibration-based WWPM studies and compare them based on the criteria listed in Table 1. Table 2 is a summary comparison of selected studies in the literature that monitor pipelines using WSNs and make use of nonintrusive sensors. As can be seen from Table 2, most of the existing WWPM studies are either centralized or decentralized since they require processing at the base station in order to detect and localize leaks.

The study of Stoianov et al. [27], referred to as PipeNet, is one of the pioneering vibration-based WWPM solutions that provide real-time leak detection. On the basis of an Intel commercial mote composed of an ARM7 core, 64 kB of RAM, 512 kB of Flash, and a Bluetooth radio for communication, a laboratory pipe rig was built to demonstrate the detection and localization of leaks using acoustic and vibration data acquired from densely spaced hydrophones and accelerometers installed along the pipeline. Local processing was performed at each node by a Fast Fourier Transform (FFT) implementation combined with a compression, while cross-correlation was implemented at the central server as the leak detection and localization algorithm. The study provides a real-time solution for leak detection, but it is not energy efficient due to the high sampling rate and processing algorithms that were used.

In [28], Sadeghioon et al. present the design and development of a multimodal Underground Wireless Sensor Network (UWSN) for pipeline structural health monitoring. They developed a sensor node consisting of a 16-bit microcontroller from Microchip, implementing their nano-watt XLP technology (PIC16LF1827), an eRA400TRS 433 MHz transceiver, two temperature sensors and one Force Sensitive Resistor (FSR) pressure sensor. According to the authors, the power consumption of the sensor nodes was minimized to 2.2 µW based on one measurement per 6 h in order to prolong the lifetime of the network. In regard to our approach, two drawbacks could be highlighted from this work: the first one is the inability to perform real time monitoring and the second one lies in the classic drawbacks from adopting a centralized approach for leak detection—reduced efficiency for a large-scale WSN, as it induces high latency and uneven energy distribution.

Martini et al. [79], in a series of tests in Bologna, used low-cost accelerometers attached to plastic pipes close to water meters in the city. They proposed to solve the problem of the high false alarm rate caused by the low accuracy of low-cost sensors and the inability of reliably detecting leakages in the midst of environmental noise, by taking measurements only during quiet times, for example, during the night when activities are reduced. However, one drawback with this approach is that does not operate in real time, as leaks cannot be immediately detected whenever they occur. Another reason is that it is difficult to find quiet times in certain areas such as city centers.

In [26], Karray et al. propose a solution called EARNPIPE which is comprised of a Leak Detection Predictive Kalman Filter (LPKF) and Time Difference of Arrival (TDOA) to detect and locate leaks. The data collected from sensors were filtered, analyzed and compressed locally with the same Kalman Filter (KF)-based algorithm. A laboratory testbed was set with plumbing components and a network was deployed, consisting of nodes composed of Arduino Due board (with an ARM cortex M3 microcontroller inside), FSR sensors used for measuring pressure and Bluetooth for communication. In this work, the high consumption of the Arduino Due board combined with the power hungriness of Bluetooth communication resulted in high consumption profiles and then a shortened lifetime for the network. The centralized approach adopted for leak detection and localization is another drawback [12].

Ismail et al., in [25,35,77], presented the development of a water pipeline monitoring system using low-cost off-the-shelf components. The experimental setup consisted of low-cost vibration sensors such as MPU6050, ADXL335 and MMA7361 sensors for the measurement of vibration occurring along the pipes, an Arduino Uno and an XBEE module for wireless transmission to a centralized decision support system. Their work showed that low-cost off-the-shelf components can be effective for leak detection in plastic pipes. Their solution was capable of distinguishing a leak from a non-leak for a leak coming from a 1-mm hole when the pressure was above 58.8 kPa. The drawbacks of this solution include the high rate of false alarms and the fact that it does not operate in real time.

In [8], Kartakis et al. presented an end-to-end water leak localization system, which exploits edge processing and enables the use of battery-powered sensor nodes. The proposed system combined a lightweight edge anomaly detection algorithm based on a Kalman filter and compression rates and a localization algorithm based on graph theory. It was validated by deploying nonintrusive sensors measuring vibrational data on a lab-based water test rig that had controlled leakage and burst scenarios implemented. The sensor nodes were based on Intel Edison development boards (embedding a dual-threaded Intel Atom CPU at 500 MHz and a 32-bit Intel Quark microcontroller at 100 MHz, 1 GB LPDDR3 POP memory as RAM and 4 GB eMMC as flash storage) and NEC Tokin ultra-high-sensitivity vibration sensors. The main drawback of this work is that the choice of commercial element (Intel Edison board) that constitutes the sensor node is not really a WSN node stricto sensu since it belongs to the Raspberry device class, with an energy efficiency and cost effectiveness that are beyond the specifications corresponding to WSN performances. In addition, they made use of a high-accuracy NEC Tokin Ultrahigh-Sensitivity vibration sensor to monitor the pipe surface vibration. However, even though our study is focused on using low-cost, low-accuracy vibration sensors for reliable leak detection, this article shows that edge computing has an emerging presence in the field.

Given the drawbacks of the solutions proposed in the literature, considering the geographic context of our deployment, which is to be done in Cameroon (a third-world developing country), and being aware of the fact that the choice of architecture and technology of the sensor node is crucial in determining its performance and power consumption, we seek a reliable vibration-based solution that operates in real time, is fully distributed, low cost, nonintrusive and also energy efficient. As a method of increasing the effective isolation of leakages in the midst of noise, we propose the use of a distributed Kalman filter (a distributed data fusion algorithm) as our leak detection algorithm.

## 4. Materials and Methods

In this section, we present a description of the off-the-shelf commercial components that make up the sensor node hardware and the leak detection algorithm we implemented.

### 4.1. Sensor Node Architecture

The architecture and technology of a sensor node is crucial in determining its cost, performance and power consumption. A fully distributed solution for leak detection in WWPM requires distributed computation, where the onboard processing capabilities of each sensor node must be capable of running the filtering, leak detection and localization algorithms. This requires the sensor nodes to operate more than just data collectors as they were initially designed for, to full-fledge information processors. Some of the main requirements of large-scale WSNs include a low power consumption and the ability to be operational for a long period of time without the replacement of the sensor node’s battery. To reduce power consumption, the computational capabilities of sensor nodes were designed to be very low, and this made nodes act as mere data collectors and wireless relays. The small physical size and low power consumption led to the development of first-generation sensor nodes [2]. The first generation of sensor nodes made use of 8-bit microcontrollers and examples include Tmote Sky, MicaZ, Mica2, Micadot, etc. [81]. The main disadvantage that makes them not suitable for deployment in our fully distributed solution is their limited computational performance (in terms of computing speed and the size of RAM and flash memory) as little or no processing of the collected data can be done onboard. The combination of 16-bit microcontrollers such as MSP430 and the CC2420 radio transceiver led to the development of second-generation sensor nodes [81] with examples such as TelosB. The second-generation sensor nodes permitted some level of storage and preprocessing at the sensor node level but are still not sufficient for a fully distributed solution. The third-generation sensor nodes were initially introduced by a generation of 32-bit microcontrollers solutions based on ARM Cortex –M0/M0+/M3/M4 and PIC32MX [46]. This was later reinforced by the emergence of the second generation of low-power 32-bit microcontrollers (led by ARM Cortex M7, dual core ESP32 and faster PIC32MZ) in the period between 2015 and 2016, and this strengthened the use of third generation sensor nodes in performing local processing [46]. Common features of this generation of microcontrollers include low power consumption, the integration of powerful digital signal processing units, support of both Wi-Fi and Bluetooth network connection and having the larger RAM and Flash memory necessary for performing complex processing on the collected data onboard [46]. The 32-bit ARM Cortex M series microprocessors have a better performance and a lower power consumption compared to the 32-bit Xtensa LX series microprocessors [46]. For example, the single-core STM32L562ME microcontroller (based on ARM Cortex M33), operating at 110 MHz, consumes less power and has a performance almost equivalent to the dual-core ESP32 microcontroller (based on Xtensa LX6) operating at 240 MHz. In addition, the ST ARM Cortex M7 microcontroller consuming 110 mA has almost five times more performance than the ESP32 consuming 68 mA [46]. A great advantage of the ESP32 is its Ultra-Low Power (ULP) coprocessor which gives it the ability to operate at ultra-low power for most WNS operations and only use the dual core when needed. Another advantage that adds to the previous one is its lower cost, which is a suitable feature for sensor nodes to be deployed in a developing country’s WWPM solution.

One example of a powerful, low-cost and low-power consumption microcontroller is the ESP32, which was released in the last quarter of 2016. This microcontroller incorporates a double-core 32-bit Xtensa LX6 microprocessor and an Ultra-Low Power (ULP) coprocessor which consumes very little power (between 10 µA~150 µA) when the main core is sleeping and can be used for basic control. This ULP feature makes it a suitable processing unit for a WSN node that will be battery powered. In addition, the ESP32 incorporates WiFi and Bluetooth modules which makes it Internet of Things (IoT) compatible. Incorporating such microcontrollers into the sensor node’s hardware, will cause the nodes to evolve from simple sensors to rich computing platforms that may even include dedicated Field Programmable Gate Array (FPGA) accelerators [44]. Our aim is to develop a high-performance, low-cost and low-power sensor node by integrating cheap and low-power off-the-shelf commercial components. Our proposed node consists of an ESP32 from Espressif Systems as the processing unit, an nRF24L01+ transceiver module from Nordic as the communication unit and an LSM9DS1 Inertia Measurement Unit (IMU) from STMicroelectronics as the sensing unit.

#### 4.1.1. ESP32

ESP32 is a low-cost, low-power System-On-Chip (SOC) increasingly used in the hobby and research development of connected embedded systems. This chip is widely used in tiny devices embedding Python or its derivatives (MicroPython, CircuitPython, etc.) for wireless embedded systems driven by a strong community such as Pycom [82] or CircuitPython [83]. This chip, although quite unused in traditional WSN hardware [44], has two main advantages: a 32 bit dual-core unit and an Ultra-Low-Power Processor (ULP) for low computation tasks. Last but not least, it presents wide support for conventional Operating Systems but also for more prospective Real-Time Operating Systems such as RIOT [84], Zephyr or Zerynth [85].

From a technical point of view, the ESP32 offers:

- for computation: an Xtensa Dual-Core 32-bit LX6 microprocessor operating at up to 240 MHz, a 520-kB Static Random-Access Memory (SRAM), a 4-MB flash memory.
- for interfacing: a 12-bit Analog-to-Digital Converter (ADC) with up to 18 channels and 40 physical General Purpose Input Output (GPIO) pads, which can be used as general purpose I/O to connect new sensors, or can be connected to an internal peripheral signal [10].
- for communication: a built-in Wi-Fi card supporting IEEE 802.11 b/g/n standards, Bluetooth version 4.2 and 486 Bluetooth Low Energy (BLE). Dedicated RF transceivers (such as nRF24L01+) can be added through GPIO to extend the RF physical layer support of ESP32 to IEEE802.15.4 protocols commonly used in the WSN community.

Engineered for mobile devices, wearable electronics, and IoT applications, the ESP32 offers advanced power management features such as Ultra-Low Power (ULP) consumption through power saving features including fine-resolution clock gating, multiple power modes, and power scaling [10]. The ESP32, when active (with the modem being off and CPU being operational), consumes currents in the [20 mA–68 mA] range and in the [10 μA–150 μA] while performing in the ULP state (only the RTC memory, RTC peripherals and the ULP co-processor are functional). The choice of this module is based on our exploration of different sensor node architectures existing in the literature [10,44,46,86]. Beyond its computation capacity coupled with an ultra-low processor, the wide coverage of the RF physical layer (Wi-Fi, Bluetooth and Zigbee ability) made the ESP32 an evident choice for our study.

#### 4.1.2. nRF24L01+

The CCXXXX (i.e., CC1000—the first generation of WSN, CC2420—the majority of WSN nodes developed in 2000s and 2010s, and CC2520—the new generation of WSN nodes) transceiver series from Texas Instruments are the most commonly used communication units in Wireless Sensor Nodes hardware development. However, we decided to use the nRF24L01+ due to its low power consumption on the one hand and for its burst mode (increased data rate) on the other hand. Even though the nRF24L01+ does not directly implement a mesh network at the MAC layer, the single dimensional aspect of our linear WSN reduces this drawback significantly.

Compliant with the IEEE802.15.4 standard at the hardware level, this transceiver operates in the (2.400–2.4835 GHz) band, exhibits current peaks in RX/TX modes lower than 14 mA (one of the lowest consumptions on the market), a sub-μA power down mode, advanced power management, and a supply voltage extending from 1.9 to 3.6 V. The nRF24L01+ is hence a true ultra-low power solution enabling months to years of battery life from coin cell or AA/AAA batteries. The burst mode is particularly interesting for distributed computing, which involves only short-distance communications between neighboring nodes and thus leads to a high data rate while keeping a low transmission power.

At the physical layer, nRF24L01+ implements Gaussian Frequency Shift Keying (GFSK) modulation, with data rates ranging from 250 Kbps to 2 Mbps. A communication range of nearly 100 m and 500 m can be achieved with and without an external antenna, respectively, at maximum power [87,88]. It is the perfect complementary RF transceiver for our node since it covers a longer range compared to Bluetooth, consumes less power than Wi-Fi and it is quite cheap from a financial point of view.

#### 4.1.3. LSM9DS1

LSM9DS1 is a nine Degrees of Freedom (DOF) IMU which features a 3D digital linear acceleration sensor, a 3D digital angular rate sensor, and a 3D digital magnetic sensor. The LSM9DS1 has a linear acceleration full scale of ±2 g/±4 g/±8 g/±16 g, a magnetic field full scale of ±4/±8/±12/±16 Gauss and an angular rate of ±245/±500/±2000 dps (degree per second). It includes an I^2^C serial bus and an SPI serial standard interface for interfacing with the microcontroller. It has an analog supply voltage ranging from 1.9 V to 3.6 V and provides an ultra-low current consumption of 600 uA when the accelerometer is in the normal mode [89]. It has three 16-bit ADCs for digitizing the accelerometer outputs which can result in more accurate digital outputs and has a wide accelerometer range for tracking both slow and fast motions. In addition to the low-power consumption feature of the LSM9DS1 IMU, another interesting feature of this sensor which makes it relevant to achieving low-power consumption is the ability of the sensor to generate an interrupt once the acceleration measured is above a predefined threshold value. With this property, the ESP32 can be programmed to operate in deep sleep mode most of the time. The LSM9DS1 will continuously monitor the pipes at all times for any deviation from the predefined threshold value. Upon deviation detection, the sensor sends an external wake-up interrupt signal to start the ESP32 main core. Thus, this threshold detection property of the LSM9DS1 makes it very useful in providing a low-power solution, as it can be used to reduce the power consumption of the sensor node and extend the lifespan of the WSN. It is also low cost.

### 4.2. Configuration of the Node

**Figure 2:**
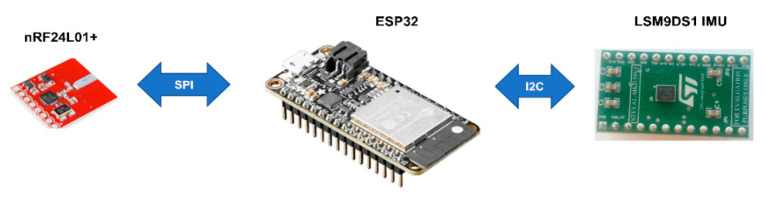
Hardware configuration of sensor node.

The nRF24L01+ transceiver module and LSM9DS1 IMU sensor are interfaced with the ESP32 via the SPI and I^2^C interfaces, respectively. The sensitivity of the accelerometer in the LSM9DS1 sensor is configured to ±2 g since this has the highest sensitivity (0.061 mg/LSB), which makes it most appropriate for detecting vibrations of smaller magnitudes such as those on the surface of a water pipe. The accelerometer collects the vibration in 3D, that, is in the X, Y and Z directions given by A_x_, A_y_ and A_z_, respectively. The magnitude of the vibration on the surface of the pipe was computed by taking the resultant acceleration in all three directions. Figure 2 represents the hardware configuration of the sensor node.

### 4.3. Leak Detection Algorithm

When a leak occurs, there is a transient change in the water pressure, which leads to a drop in the internal pipe pressure and an increase in the pipe surface vibration [34,60]. The pipe surface vibrations resulting from the sudden drop in pressure can be detected by an accelerometer. However, since low-cost sensors are used in our case study, their readings might be inaccurate and also be drowned out by noise from the surrounding and operational conditions such as the opening/closing of pumps, valves, etc. For this reason, an appropriate leak detection algorithm is required for processing the measured vibration data to improve on the accuracy of detecting leaks. To maximize leak detection and minimize the number of false alarms without involving any form of centralized computation, we proposed the use of a Distributed Kalman Filter (DKF), which is simply a combination of a local Kalman filter and distributed data fusion, as the leak detection algorithm. In this sub-section, we explain the reason for our choice of this algorithm and the implementation of the algorithm.

#### 4.3.1. Reasons for the Choice of Leak Detection Algorithm

Price often has a direct bearing on the quality of a node’s sensors and influences the accuracy of the result that can be obtained from a single node [90]. Thus, using low-cost nonintrusive sensors in WWPM to detect leak signals is usually characterized by inaccurate measurements and may result in false alarms in the leak detection system. According to Figure 1, the computational algorithms used for processing and analyzing leak signals from field sensors in order to detect the presence of leaks on a WDN can be categorized into signal processing, model-based and data-driven algorithms. The signal processing algorithms extract information from the measured data and compare it with data sets from a fault-free benchmark so as to detect the presence or absence of a leak. Most signal processing algorithms analyze data in the frequency domain, and thus require mathematical conversion [91]. This makes them computationally intensive and will result in huge power consumption when implemented on sensor nodes. The model-based methods usually involve the use of mathematical functions or formulas to represent or replicate the operation of a WDN. They can determine the approximate leakage location by comparing pressure or flow measurement with their estimation obtained using the hydraulic network model [60]. The drawback with the model-based methods is that they require a precise mathematical model of the pipeline system in order to accurately detect leaks. With the data-driven methods, large amounts of data are being collected and used to analyze, interpret, and extract useful information for operational and other purposes based on Artificial Intelligence (AI) techniques and other data-driven methods [61]. Their drawback is that they need a large amount of data and a long training time [61].

In recent studies such as Karray et al. [26] and Kartakis et al. [8], the Kalman filter has been used as a signal processing algorithm for filtering the leak signals obtained from nonintrusive sensors installed on the pipeline in order to detect leaks. The Kalman filter analyzes data straightforwardly in the time domain, which makes it computationally less intensive and thus suitable for implementation on a sensor node. The study of Karray et al. [26] is a centralized solution while that of Kartakis et al. [8] is a decentralized solution for leak detection in WWPM. However, our work is focused on providing a fully distributed solution for leak detection in WWPM and for this reason we propose the use of a Distributed Kalman Filter (DKF). To the best of our knowledge, this is the first work that uses WSNs with nonintrusive sensors and a DKF for leak detection in WWPM. The idea is to provide a solution that will improve the reliability of leak detection and also provide a fully distributed solution that curbs the limitations of centralized solutions, i.e., high latency (due to multi-hop communication), scalability issues and high power consumption. In our proposed solution, each sensor node runs a local Kalman filter to obtain an accurate local estimate from the local measurements, then later fuses it with those of its neighbor to achieve a more accurate global estimate used for leak detection. In this way, our proposed solution is autonomous and does not need any central intelligence.

In [37], He et al. did a review on a number of DKF algorithms for low-cost sensor networks. According to their classification, DKF algorithms can be classified as either sequential, consensus, gossip, or diffusion, based on how local sensor nodes communicate with their neighbors to carry out data fusion. The DKF algorithm, which we implemented, was proposed by Battistelli et al. [58] and falls into the category of diffusion-based DKF algorithms according to the study of He et al. [37]. The algorithm is fully distributed, robust and achieves local consistency. Another advantage of this algorithm is its event-triggered communication capability (which reduces the communication burden), as the nodes will only transmit their local estimate with their close neighbors when the difference between the present local estimate and the last transmitted local estimate is above a certain threshold. This threshold determines the transmission rate and it is a parameter that can be varied. This can be used to make the WSN energy-aware and also energy efficient as nodes can regulate their transmission rate and thus their energy consumption based on their present energy (battery) level. Nodes with high battery levels can have a higher transmission rate and thus have more accurate estimates while nodes with low battery levels can transmit less and thus have less accurate estimates. In this way, the WSN can be adaptive.

#### 4.3.2. Brief Description of the Kalman Filter Algorithm

The Kalman filter is an information filtering technique for filtering information known to be prone to error, uncertainty, or noise. The goal of the filter is to take in this imperfect information, sort out the useful parts of interest, and reduce the uncertainty or noise [92]. There are two types of noise associated with stochastic estimation, process noise and measurement noise. Process noise can be explained as the difference between the real system and the model, while measurement noise is the noise associated with the sensors and instrumentation. The Kalman filter minimizes the estimated error covariance in a linear stochastic system, has low memory requirements and low complexity [93], and it is capable of handling situations with a lot of noise or high uncertainty in the data. This therefore makes it a good candidate for improving the accuracy of noisy measured leak signals and detecting leaks in WWPM, as nodes are limited in their memory, processing power and energy [8,61,93].

The Kalman filter is based on two steps, comprising a prediction followed by a correction to determine the states of the filter. This is sometimes called predictor–corrector, or prediction–update [92].

In the first step, the estimated state x, at time k, is predicted from the updated state at time k-1. The prediction of the current state and the covariance matrix is given by [92,93]:

(1) $${\hat{x}}_{k}^{-}=A{\hat{x}}_{k-1}+Bu_{k}$$

(2) $$P_{k}^{-}=AP_{k-1}A^{T}+Q_{k}$$

where ${\hat{x}}_{k}^{-}$ is the predicted state vector at time *k*, ${\hat{x}}_{k-1}$ is the previous estimated state vector, $P_{k}^{-}$ represents the predicted state error covariance matrix, *A* and *B* are matrices defining the system dynamics, $u_{k}$ is the input vector, $P_{k-1}$ is the previous estimated state error covariance matrix, and *Q* is the process noise covariance matrix.

The second step is the correction or update step. This step aims to get an improved estimate by incorporating new measurements into the predicted estimate using the Kalman gain (*K_k_*).

(3) $$K_{k}=P_{k}^{-}H^{T}{\left(HP_{k}^{-}H^{T}+R_{k}\right)}^{-1}$$

(4) $${\hat{x}}_{k}={\hat{x}}_{k}^{-}+K_{k}\left(z_{k}-H{\hat{x}}_{k}^{-}\right)$$

(5) $$P_{k}=\left(I-K_{k}H\right)P_{k}^{-}$$

where *H* is a matrix necessary to define the output equation, *R* is the measurement noise covariance, *I* is an identity matrix, ${\hat{x}}_{k}$ is the estimated or updated state vector, $z_{k}$ is the measurement at time *k* and $P_{k}$ is the updated state error covariance.

#### 4.3.3. Distributed Kalman Filter Algorithm Implementation

Distributed Kalman Filter (DKF) algorithms have been used extensively in low-cost WSN-based target tracking applications [37]. They can be used in any application where it is required to improve the accuracy of a monitored parameter by using redundant information from multiple low-cost sensors to effectively complement the limitations of a single sensor node. They can be extended to applications such as navigation systems, environmental and power system monitoring, autonomous robot systems, large-scale camera networks, wireless channel monitoring, etc.

A number of DKF algorithms have been presented in the literature [37,58,94,95]. In our solution, we implemented the diffusion-based DKF algorithm proposed by Battistelli et al. [58] as a starting point to establish the importance of distributed data fusion in improving the leak detection accuracy. Their study presented a novel event-triggered distributed state estimator based on a consensus Kalman filtering approach, as well as a transmission triggering condition which essentially requires that the local estimate and/or covariance of a given node be sufficiently far away from the ones computed by neighbors before there can be an exchange of data between a node and its neighbors. The paper addresses Distributed State Estimation (DSE) over a network where each node can process local data as well as exchange data with neighbors. In their proposed DSE algorithm, each node runs a local Kalman filter and then, in order to improve its local estimate, fuses the local information with that received from its in-neighbors [58].

**Figure 3:**
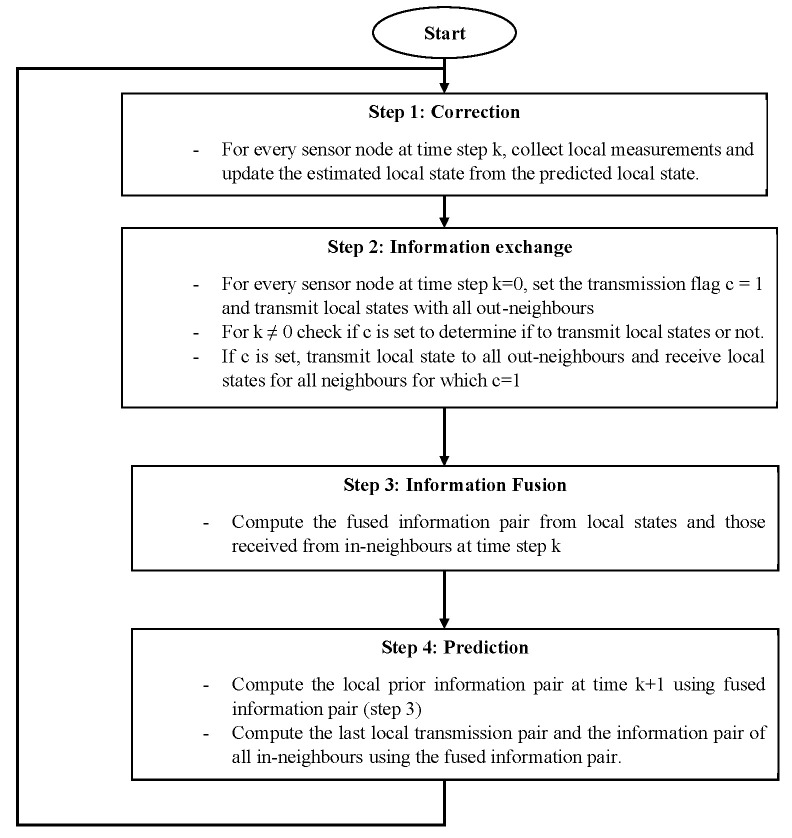
Flow diagram of implemented distributed Kalman filter algorithm.

The DKF algorithm proposed by Battistelli et al. [58] consists of four main steps (correction, information exchange, information fusion and prediction) and every sensor node implementing the algorithm goes through the iterative process shown in Figure 3. Each node starts by updating a local information pair, which consist of the local estimate and the estimation error covariance matrix. This is immediately followed by the exchange of the information pair to the out-neighbors of each node if and only if the transmission flag of the sensor node is set to 1. The transmission flag is set to one when the difference between the current updated local estimate and the last transmitted local estimate exceeds some threshold, which is determined by some transmission parameters designated in the study as α, β, and δ (which can be varied to achieve a desired behavior in terms of transmission rate and performance). By this, the algorithm possesses an event-triggered communication capability and information is only shared when the data currently computed by a node’s out-neighbors are no longer consistent with the data locally available at the node. The transmission test ensures that in the case of no transmission, the data currently computed by the out-neighbors of a node are close to the data locally available at the node, both in terms of mean and covariance, thus maintaining local consistency. The information exchange step is then followed by an information fusion step where every node computes a fused information pair from its local information pair and those received from in-neighbors at the current time step, k. During the information fusion step, each node computes an approximate local pair for its in-neighbors that did not transmit at time step k (because their transmission flag was not set), from the most recent local information pair last received from them. As the final step in each iteration, the prediction step involves propagating the fused information pair in time by applying the Kalman filter prediction step to compute the local predicted information pair at time k + 1.

In our implementation, A and H in the model equations are constants since we are dealing with a one-dimensional Kalman filter. H is one because it is known that the measurement is composed of the state value and some noise, while A is one because it is assumed that the next value will be the same as the previous one. We derived R from the LSM9DS1 datasheet. The linear acceleration typical zero-g level offset accuracy given in the datasheet is ±90 mg, thus R is 0.0081. Q is obtained after some experimentation. From the datasheet, the sensor in a steady state on a horizontal surface will measure 0 g on both the *X*-axis and *Y*-axis, whereas the *Z*-axis will measure 1 g. We did some experiments with different *Q* values (10, 1, 0.1, 0.01, and 0.001) and selected the one that best approximated the acceleration values at zero-g. Q, equal to 0.001, best approximated the zero-g acceleration values.

For the physical implementation, the proposed DKF algorithm was written in C and the sensor nodes having the Adafruit feather ESP32 (Huzzah32) microcontroller as the processing unit, were programmed using the Arduino 1.8.9 Integrated Development Environment (IDE) according to Adafruit recommendation. The RF24 [96] and RF24Network [97] libraries, which provide the MAC and Network layer functions were used to control the nRF24L01+ transceiver interfaced to the Huzzah32 via SPI. The firmware uploaded to the nodes after compiling the proposed DKF algorithm using the Arduino IDE occupied a storage space of 225 kB.

In our preliminary study, which demonstrates how a distributed Kalman filter (a distributed data fusion technique) can improve the reliability of leak detection in low-cost vibration-based WWPM systems, the proposed DKF algorithm [58] was implemented on a network of two nodes, with addresses given by 00 and 01 (octal representation) and the node 00 being the base node or Personal Area Network (PAN) coordinator. This is because, in this study, we are dealing with a linear WSN, which is the case of a WSN-based water pipeline monitoring system, as each sensor node has a maximum of two directly connected neighbors. The ideal is to have a sensor network of at least three sensor nodes to demonstrate distributed data fusion in a linear WSN. However, a linear WSN consisting of two sensor nodes provides a firsthand yet precise evaluation of performance, since in a linear WSN, the beginning and ending nodes of the chain have just one single directly connected neighbor. In addition, the parameters that determine the information transmission rate of the proposed DKF algorithm, represented in [58] as α, β, and δ, were given the values 0.001, 40 and 40, respectively, in our implementation.

## 5. Experimental Setup

### 5.1. Simulations

The implementation and deployment of a WSN incurs cost and it is also time consuming. So, it is important to simulate the operation of a specific design before deploying it. We performed simulations to evaluate the performance of the proposed algorithms. Simulations were carried out in Cupcarbon 4.2, which is a Smart City and Internet of Things Wireless Sensor Network (SCI-WSN) simulator that is used to design, visualize, debug and validate distributed algorithms for monitoring, e.g., the collection of environmental data [98]. It offers two simulation environments; one enables the design of scenarios with mobility and the generation of natural events and the other enables the simulation of discrete events in WSNs. CupCarbon simulation is based on the application layer of the nodes. It is composed of four modules: a microcontroller, radio unit, sensing unit and a battery [99]. It also includes a script called SenScript, which allows us to program and to configure each sensor node individually. From this script, it is also possible to generate codes for hardware platforms such as Arduino/XBEE [99]. This feature makes it suitable for simulating distributed algorithms in a WSN environment and the ability to demonstrate distributed computing in a WSN, since it permits us to write and simulate applications that will be implemented on real sensor nodes. In addition, in CupCarbon, the energy consumption can be calculated and this allows us to represent the detailed energy profile of each sensor node. This allows us to test the feasibility and realistic implementation of a network before its real deployment. This particular feature of CupCarbon is important to our work, as we are interested in monitoring the energy consumption of the nodes in our proposed distributed solution.

**Figure 4:**
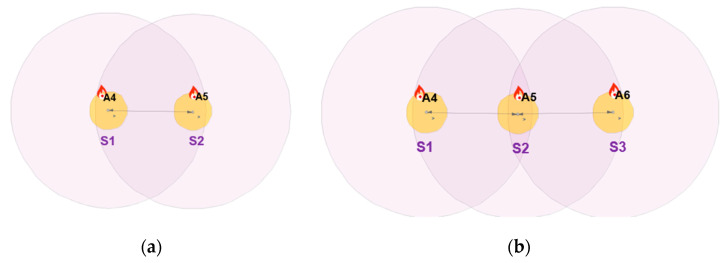
Simulation setup in CupCarbon: (**a**) with two nodes; (**b**) with three nodes.

The simulation setup shown in Figure 4a is composed of two sensor nodes (S1 and S2) and natural event generators (A4 and A5). The natural event generator enables the generation of analog values and its objective is to simulate random or given values from the environment. The simulation setup shown in Figure 4b consists of three nodes (S1, S2 and S3). The simulation depicted in Figure 4b was carried out to show that the results of the simulation of the DKF algorithm in a linear network composed of two nodes and a linear network composed of three nodes are similar. A natural event was used to simulate acceleration values read by the LSM9DS1 IMU sensors. One thousand acceleration values generated by the natural event were used in the simulations.

Two scenarios were then simulated using the linear WSN consisting of two sensor nodes. In the first case, we simulated a scenario where the data measured by both nodes (S1 and S2) were erroneous (noisy) while in the second case, only the data measured by one of the nodes (S1) were erroneous and the data measured by the other node (S2) were error free. For each case, we implemented both the local Kalman filter (without data fusion) and the DKF (with data fusion) and compared the results. The simulation results will be discussed alongside the experimental results in Section 6 so as to validate the approach.

### 5.2. Laboratory Testbed Setup

**Figure 5:**
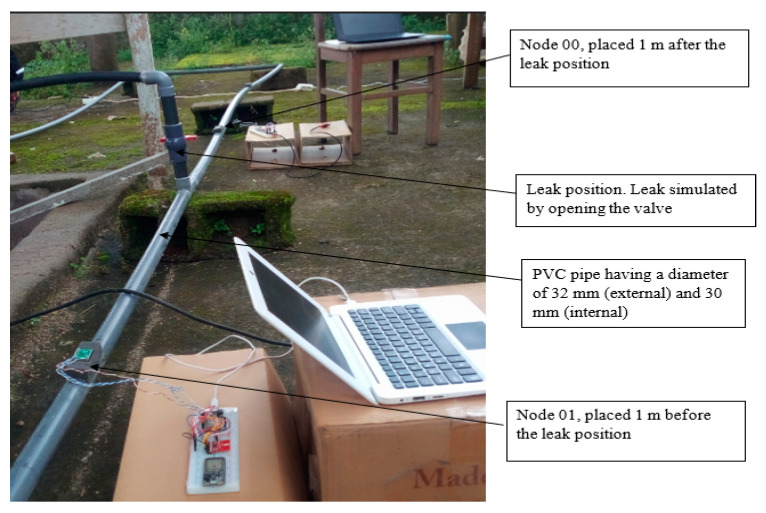
Setup of the laboratory testbed.

A laboratory testbed was installed in the Electrical and Electronic Laboratory of the University of Buea, Cameroon, based on technical and real-field observations of WDN in Cameroon. The installation is composed of two plastic water storage tanks of capacity 1000 L (one tank for storage placed on a tower of height 9 m and one supply tank placed beneath the tower), a U-shaped 13-m long PVC pipe with an external diameter of 32 mm and an internal diameter of 30 mm for the plumbing part. This installation distributes water by using an electrical pump (0.7 HP motor) providing a maximum pump capacity of 40 L/min to fill the upper storage tank. Leakage emulation in the pipeline is realized by a valve (size of the leak is controlled by the opening of the valve) situated 4 m away from the inlet of the water into the system. Figure 5 depicts the testbed setup.

Our WSN experimental setup consists of two sensor nodes, namely Node 00, placed one meter after the leak position and Node 01, placed one meter before the leak position, as shown in Figure 4b. The IMU sensors provide vibration measurements through acceleration monitoring.

To measure the performance of our leak detection solution using DKF algorithm, we emulated a leak at a single location along the pipeline, as shown in Figure 5. In this deployment, the leak location is fixed, but variations in the sensors can be made to evaluate the effectiveness of our solution. We carried out measurements for two scenarios: a local Kalman filter implementation and a DKF implementation. For these experiments, we stored traces of data collected from the two sensors, and compared the effectiveness of the approach, i.e., the effect on leak detection when the sensor nodes implemented the DKF algorithm and in the case where only a local Kalman filter was implemented.

### 5.3. Power Consumption Measurement

In this sub-section, we describe the development of a power measurement device we call a USB power meter that we used to measure the power consumption of the sensor node in order to establish the energy profile of our distributed solution. We developed this device for two main reasons: (1) to permit us to measure very low currents in the µA range in order to measure the consumption of the node when the ESP32 is operating in deep sleep mode (i.e., when the ULP coprocessor is in control) and (2) to be able to monitor and store the power consumption of the nodes without physically being present (i.e., recording of power measurements collected periodically over a long period of time).

Current consumption monitoring is a very important aspect for battery-powered sensor nodes since they are constrained in terms of energy. Selecting the correct method to monitor the current consumption of a sensor node is critical in optimizing the system performance. There are three primary approaches used to profile the power of systems and components. They include simulator-based power estimation, direct measurements, and event-based estimation [100]. We make use of the direct measurement method which can be done via operational/difference (milliamps to tens of amps), instrumentation (nanoamps to tens of amps), or current sense (tens of microamps to tens of amps), and where power can be directly measured both intrusively or nonintrusively. The intrusive measurements require inserting precision (shunt) resistors into the power supply lines of components under study and use power meters to measure the voltage drop across the resistor. The current through the component is calculated by the voltage drop over the shunt resistor divided by its resistance. The nonintrusive approach uses ammeters to measure the current flow of the power supply lines directly [100]. Since the currents we are measuring are in the range of tens of microamps to amps, we used the current sense method. The current sense device we used was the INA226.

**Figure 6:**
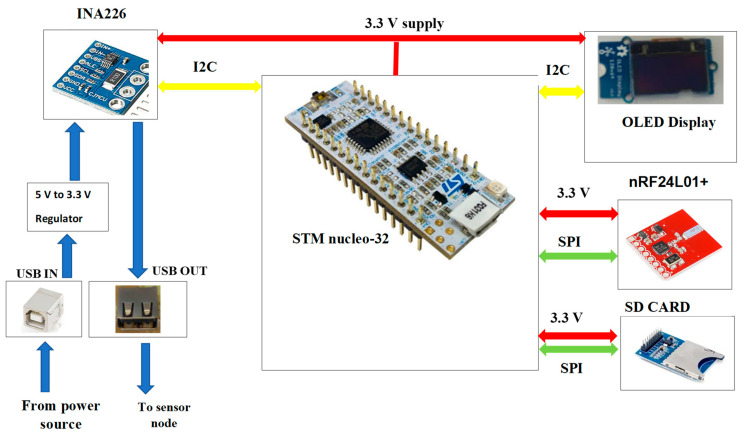
Block diagram of power measurement device.

The power measurement device developed for power consumption of the sensor node is based on the intrusive direct measurement method and is composed of a 100 mΩ shunt resistor, INA226 module, STM nucleo-32 F303k8 microcontroller, nRF24L01+ transceiver, 128 × 64 OLED display, SD card and two USB ports. Figure 6 is a block diagram display of the USB power meter.

The INA226, often used in instrumentation for low current monitoring, is a current shunt and power monitor with an I^2^C compatible interface. It was used to monitor both the shunt voltage drop across the shunt resistor (placed in series with the sensor node) and the bus supply voltage. The INA226 can be used either in high-side sensing (where a shunt resistor is placed between the supply voltage and the load) or low-side sensing (where a shunt resistor is placed between the load and the system ground). High-side sensing was used since it is preferable when dealing with low currents given that it is more responsive to changes in the current flow and it adds no disturbance to system ground [101]. The INA226 is designed for a maximum input shunt voltage of 81.92 mV and has a 16-bit ADC. Thus, the maximum current that can be measured by the device is 819.2 mA and the resolution is 25 µA in the case where a 100 mΩ shunt resistor is used. Using the 100 mΩ shunt resistor permits us to be able to measure the current consumption of the ESP32 when it is in the deep sleep mode (with currents in the µA range). In addition, the voltage drop across the shunt resistor allows sufficient voltage to power the ESP32. The STM nucleo-32 F303k8 interfaces with the INA226 and also configures its programmable calibration value, conversion time and averaging mode. For better accuracy in measurements, the conversion time and averaging mode were configured to 140 µs and 4, respectively. The OLED display is for displaying the power measurements, the SD card is for storing the power measurements over a long period of time and the nRF24L01+ transceiver permits the remote reading of the power consumption. USB-IN is used to supply power to the measurement board while USB-OUT is there to power the sensor node.

## 6. Results and Discussion

In this section, we present and discuss the simulation and laboratory results. In addition, the results of the power consumption obtained via simulations and physical measurements are also presented.

### 6.1. Scenarios

This sub-section presents the scenarios used in both simulations and field experiments to validate our proposed approach.

Two scenarios were simulated. In the first scenario, we simulated a case where the data measured by both nodes (S1 and S2) were erroneous (noisy) to the same extent, i.e., the measurement noise of both sensors is correlated while in the second scenario, only the data measured by one of the nodes (S1) were erroneous while the data measured by the other node (S2) were error free, i.e., the measurement noise of both sensors are uncorrelated. For each scenario, we implement both the local Kalman filter (without data fusion) and the DKF (with data fusion).

For the field experiments, we carried out measurements for two scenarios: a local Kalman filter implementation (i.e., Kalman filtering requiring no fusion of estimates with neighboring node) and a DKF implementation (i.e., Kalman filtering requiring fusion of estimates with neighboring node).

### 6.2. Validity of Approach on a Two-Node Linear Wireless Sensor Network

In a chain of sensor nodes forming a linear WSN, the nodes at the beginning and ending of the chain have just one directly connected neighbor, while all the intermediate nodes have two directly connected neighbors each. To demonstrate distributed data fusion in a linear WSN, an ideal case is to have a network of at least three nodes, as it represents all the relationships that can be found in a larger linear WSN. Our laboratory testbed only enables us to perform a two-nodes physical evaluation. For this reason, we restrained our first approach to two nodes for simulation and physical experimentation.

**Figure 7:**
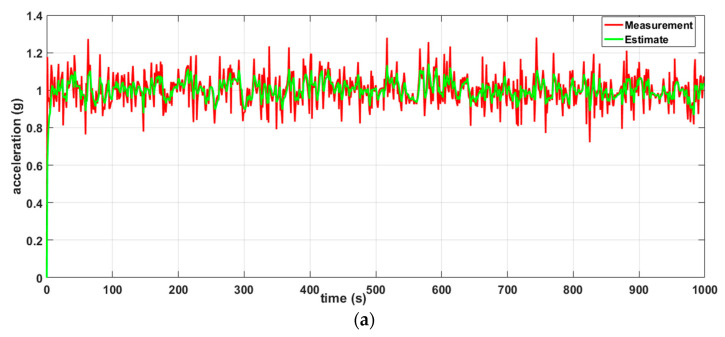

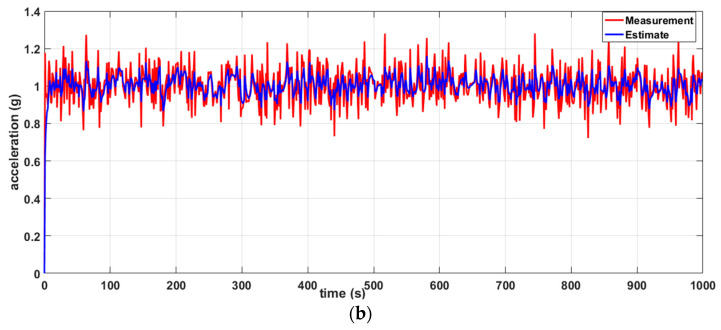
Results of simulation the distributed Kalman algorithm: (**a**) two-node linear Wireless Sensor Network (WSN); (**b**) three-node linear WSN.

**Figure 8:**
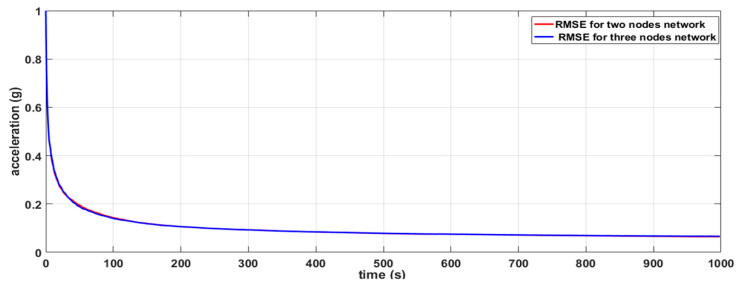
Comparison of root mean square error (RMSE) for node S1 in the case of two-node and three-node linear WSN.

To validate that this two-nodes approach is still valid, we simulated a DKF algorithm implementation on a two-node linear WSN (Figure 7a) and three-node linear WSN (Figure 7b). Figure 8 depicts a comparison of the root mean square error (RMSE) of node S1 when the simulations were performed in both two-node linear network and three-node linear network. The RMSE of S1 in the two-node linear network converges to 0.064 while it converges to 0.066 in the three-node linear network. From these results that confirm the strong similarity between Figure 7a,b, we can infer that a linear WSN consisting of two sensor nodes can be used to validate the feasibility and performance evaluation at the node level. We will continue our analysis on a linear WSN comprising of two nodes.

### 6.3. Comparison of Results from the Two Simulation Scenarios

In this sub-section, we present and discuss the results of vibration estimates obtained for the simulation scenarios described in Section 6.1.

**Figure 9:**
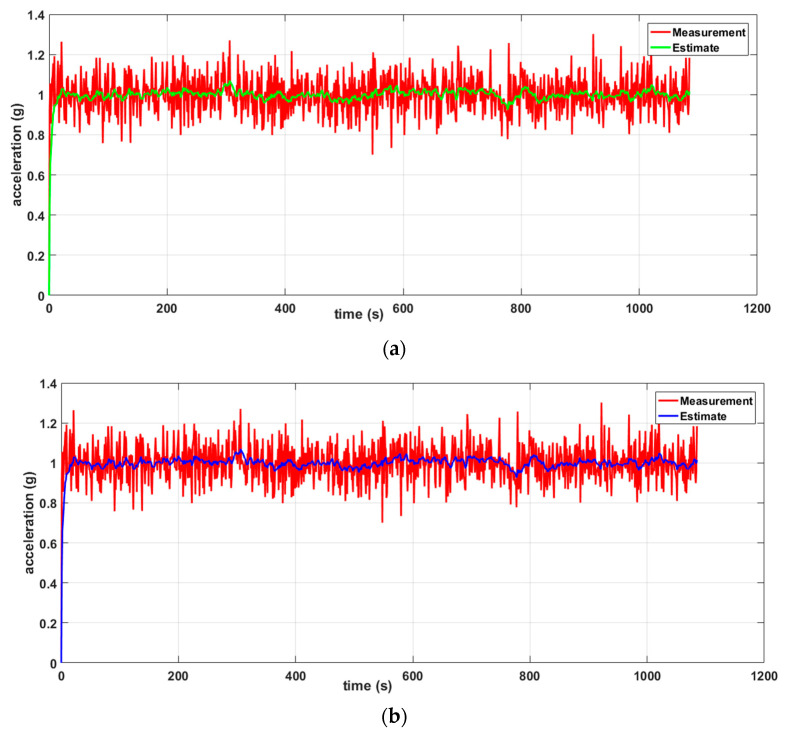
Results for distributed Kalman filter implementation with noisy measurements from both sensor nodes. (**a**) Sensor node S1. (**b**) Sensor node S2.

**Figure 10:**
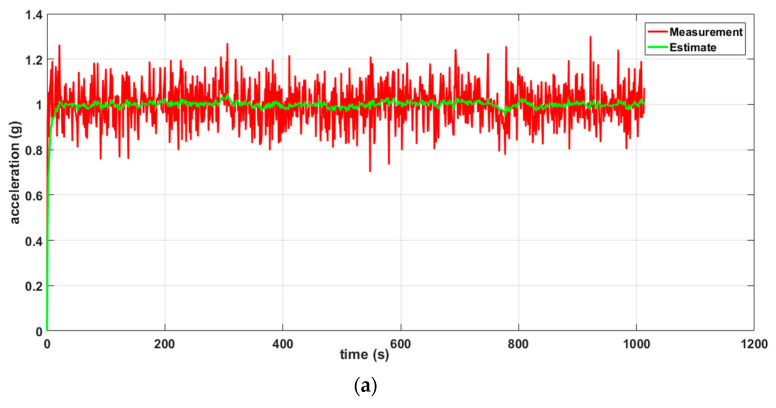

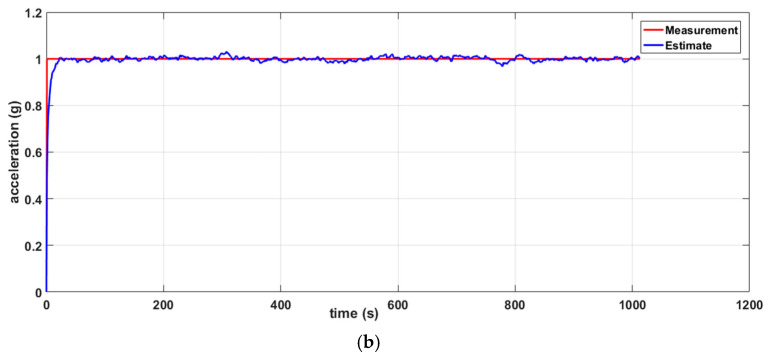
Results for distributed Kalman filter implementation with noisy measurements from a single sensor node. (**a**) Sensor node S1. (**b**) Sensor node S2.

Figure 9 and Figure 10 show the results obtained from simulations in CupCarbon 4.2 when the DKF algorithm was implemented on both nodes (S1 and S2). Figure 9 illustrates the results obtained for the scenario where the measurements of both sensors are correlated (noisy to the same extent) while Figure 10 illustrates the results obtained for the scenario where the data measured by both sensor nodes are uncorrelated (measurements from the sensor node (S1) are erroneous and that of the other sensor node (S2) are error free).

From the results depicted in Figure 9, it can be seen that there is not a great improvement in the estimates after filtering and fusion, since the measurements of both nodes are noisy to the same extent in this scenario. This is because when the number of incorrect data sources are greater than the number of correct data sources, the overall performance of the fusion process can be reduced [38]. In Figure 10, there is a great improvement in the estimates of the sensor node (S1) with noisy measurements, since it fuses its local estimate with the local estimate of sensor node S2 with noise-free measurements. The results of sensor S1 in Figure 10 are closer to the true value compared to those in Figure 9. When the local Kalman filter algorithm was implemented, there was no difference in the results obtained from both scenarios. There was no improvement in the estimates of S1 even when its close neighbor S2 had error-free measurements. This is because there is no fusion of local estimates from neighboring nodes in the local Kalman filter implementation. This result establishes the importance of distributed data fusion in improving accuracy in a fully distributed solution. We validate this assertion via physical measurements in Section 6.3 where we implement both the local Kalman filter and DKF algorithm for leak detection in a laboratory WDN and compare their performance.

### 6.4. Comparison of Results from the Two Experimental Scenarios

In this section, we present and discuss the results obtained for the physical experimentation scenarios described in Section 6.1 so as to validate the simulation results presented in Section 6.2.

**Figure 11:**
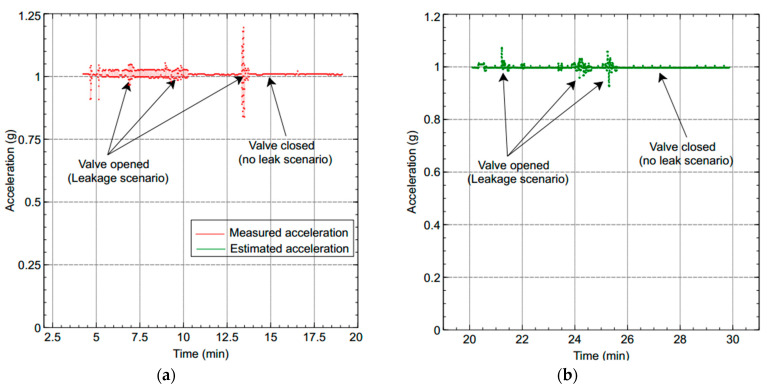
Estimated acceleration of Node 00 obtained from Distributed Kalman Filter (DKF) implementation (**a**) with measured acceleration and (**b**) without measured acceleration.

**Figure 12:**
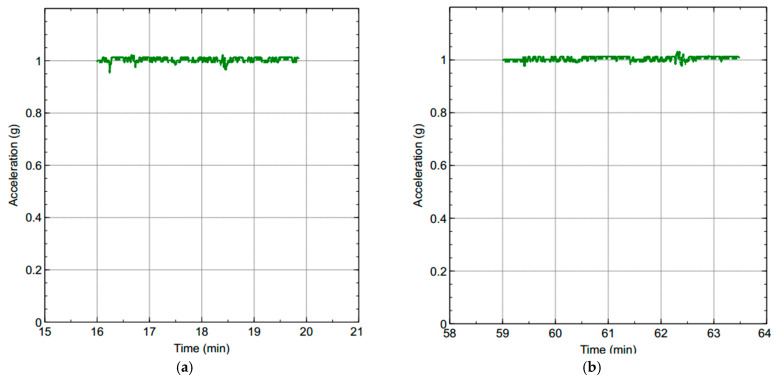
Estimated acceleration obtained from local Kalman filter implementation: (**a**) Node 00; (**b**) Node 01.

Figure 11 and Figure 12 are results obtained from the laboratory testbed. Figure 11 represents the data obtained from Node 00 when the DKF algorithm was implemented on both sensor nodes while Figure 12 represents the results obtained when only a local Kalman filter was implemented on both sensor nodes without distributed data fusion.

When there is no leakage, the measured acceleration on the pipe surface is 1.00 g while the estimated acceleration on pipe surface after performing Kalman filtering is 0.99 g. As shown in the results obtained in the field (Figure 11), the estimated acceleration of the pipe when there is no leakage is below 1.01 g while an estimated acceleration greater than 1.01 g corresponds to a leakage on the pipe. This is because when there is a leak, there is a fast drop in pressure, leading to an increase in the flow turbulence which is significantly responsible for the vibrations of the pipe walls, since the source of vibration is dissipated energy caused by turbulence.

Comparing the results in Figure 11 (where we implemented DKF) with those of Figure 12 (where we implemented only local Kalman filter), reveals that we can easily isolate a leakage scenario from a non-leakage scenario in the case where we used DKF. This increases the performance or reliability of detecting leaks and minimizes the rate of false alarms. However, it was difficult to distinguish a leakage scenario from a non-leakage scenario when we used only a local Kalman filter. The data depicted in Figure 12 have a higher likelihood of producing false alarms since the estimated acceleration computed by the local Kalman filter still has a lot of uncertainties. As shown in Figure 12, the estimated acceleration is fluctuating rapidly over short time periods. Applying the fixed threshold acceleration of 1.01 g will result in a higher rate of false alarms. This leads to multiple alarms and associated alarm clears as an alarm is declared each time the estimated acceleration fluctuates above the threshold value of 1.01 g and as it fluctuates back below the threshold, the alarm clears.

Examining the results from simulation and physical deployment, we can draw the conclusion that with the proposed DKF algorithm implemented, there is a bit of improvement in the reliability of leak detection compared to when the local Kalman filter is implemented. This improvement can be attributed to the distributed data fusion capability of the DKF algorithm. However, one very challenging issue for distributed algorithms in a WSN is their robustness to failed transmissions during the communication process. The results obtained in simulations assumed that no messages were lost during the communications between neighboring sensor nodes and also did not account for delays due to packet loss. Notwithstanding, the results obtained from simulations are very close to what we observed from the physical experimentation. We also observed that in the physical implementation the packet loss rate was very low (<5%). This can be explained by the fact that, as a result of distributed computing, the number of communicating nodes for each node is limited to just the directly connected neighbors and which when applied to our context of a linear WSN limits the number of directly connected neighboring nodes to an upper bound of 2. By properly making use of the transmission time schedule capability of the RF24Network [97] library, the number of collisions is significantly reduced, thus reducing the transmission rate. In addition to this is the event-triggered capability of the proposed DKF algorithm [58] that we implemented in this our preliminary study. The event-triggered nature of the proposed algorithm reduces the packet transmission rate which also reduces the packet loss rate. From the results presented by the authors in [58], it is clear that the transmission rate of the proposed algorithm is not uniform over time [58]. The proposed algorithm has a higher packet transmission rate at the beginning when the estimation error is large. The packet transmission reduces when the estimation error is low due to consistency in local estimates and increases again in correspondence with variations in the monitored parameter.

### 6.5. Power Evaluation of Proposed Distributed Kalman Filter Solution

In this sub-section we present the power profile of our proposed DKF solution obtained from the simulations and physical measurements. In addition, we also present results from physical measurements that show how the power consumption of the solution can be greatly reduced by using the ULP coprocessor of the ESP32 microcontroller serving as the processing unit of the sensor node.

#### 6.5.1. Simulation of Power Consumption

**Figure 13:**
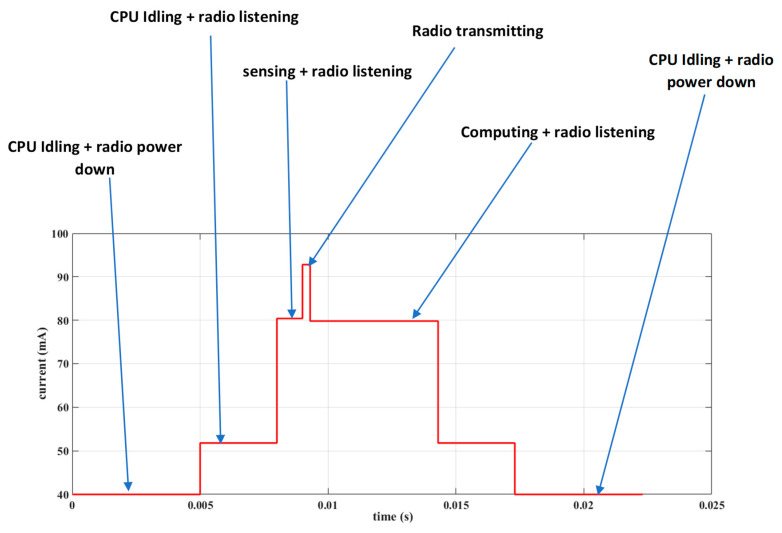
Sensor node’s current profile from simulation.

We used the current consumption data from the datasheets of the different components (ESP32, nRF24L01+, and LSM9DS1) that constitute our sensor node. These data are used in CupCarbon to emulate the power consumption of the sensor node when operating in the different states (sensing, transmitting, computing, receiving, sleeping). Figure 13 shows the current profile of the sensor node derived from simulations when the ESP32 is operating at 240 MHz. This is what is used by the simulator to compute the power consumption of each sensor node when each of the distributed algorithms is being simulated. With this, we can have a datasheet-based energy model of the power consumption of nodes embedding every selected distributed algorithm we are going to study before physical implementation. We used the physical implementation to validate the results of the power consumption obtained from simulation. In addition, the state of charge of the battery of each sensor node can be obtained from CupCarbon. This enables the identification of critical nodes and can give us information on the lifetime of the sensor network when a given distributed algorithm is being implemented. This provides an initial revelation of which algorithm is more power efficient. Finally, from simulations in CupCarbon, we can also obtain information of the number of messages transmitted which reveals the bandwidth consumption of a given distributed algorithm.

To evaluate the energy budget of the node per cycle, we established Table 3 from the datasheets of our sensor node’s contituent components.

As shown in Table 3, the sensor node will consume most its battery energy when it is listening for packets. Since we are dealing with a realtime application, where the loss of packets needs to be minimized, the radio has to be continuously listening for packets. In Section 6.5.3, we present a corresponding table of the node’s energy consumption at different states, obtained from measurements, and compare it with those obtained from simulations.

#### 6.5.2. Power-Consumption Measurement on Laboratory Testbed

**Figure 14:**
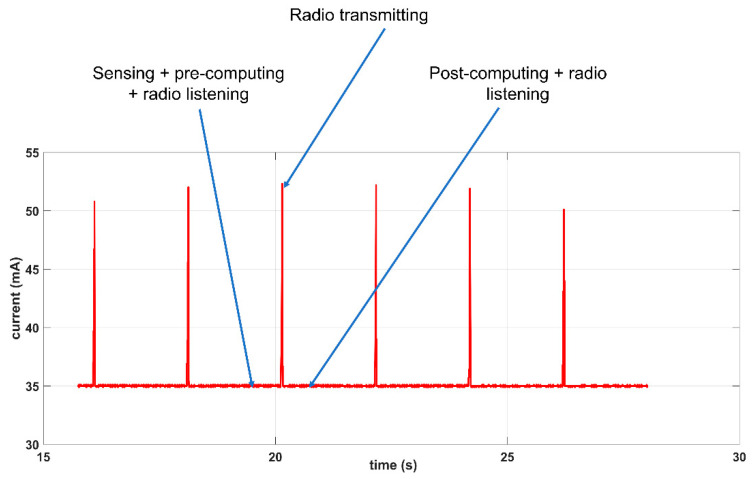
Sensor node’s current profile from physical measurements.

We performed power consumption measurements on the nodes of our real laboratory testbed. The average power consumed by the node, as depicted by Figure 14, is about 100 mW (35 mA at 3.3 V), which is relatively very high for an application that will be powered by a battery. The high power consumption is explained by the fact that our current implementation uses the ESP32 in modem sleep mode (with current consumption in the range 20–31 mA at 80 MHz clock speed) and the radio was always in the listening mode throughout. In modem sleep mode, the ESP32 is actually a power-hungry chip and, as such, is not suitable for battery-powered sensor nodes. One way of reducing the power consumption of the sensor node is by reducing the amount of time for which the main core of the ESP32 is active by preferably using the ULP coprocessor for basic control.

**Figure 15:**
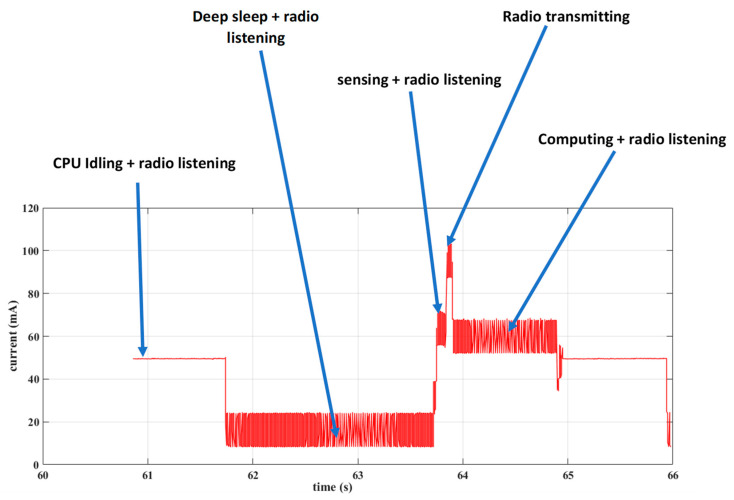
Sensor’s node current profile with deep sleep implemented.

In order to reduce the node’s power consumption, we decided to harness the ULP coprocessor of the ESP32 by putting the node frequently into deep sleep mode (with current consumption in the range 10–150 µA). Thus, the node will only be active for very short periods of time, when it needs to transmit data and perform fusion. Figure 15 illustrates the results obtained from putting the ESP32 main core into deep sleep mode while using the ULP coprocessor. The ESP32 main core was programmed to sleep for 2 s while the ULP coprocessor was functional and it was awakened from sleep using an internal interrupt. In this demonstration, we used the timer interrupt, which awakened the ESP32 main core when the 2 s sleeping period expired.

As shown from in the results displayed in Figure 15, when the node is in deep sleep mode, the measured current consumption of the node can be as low as 11.8 mA, which corresponds to the current consumed by the nRF24L01+ transceiver in listening mode (11.8 mA), the LSM9DS1 IMU when the accelerometer is operational (600 µA) and the current consumed by the ESP32 in deep sleep mode (10 µA~150 µA). Continuously putting the node into deep sleep mode and only waking it up and keeping it awake for short periods of time to perform transmission and data fusion with neighboring nodes will greatly reduce the power consumption of the node and increase the lifespan of the WSN. The node only wakes up when it receives an external interrupt from the nRF24L01+ (i.e., when it receives data from a neighboring node) or when it receives an external interrupt from the LSM9DS1 (i.e., signifying the threshold acceleration value has been exceeded and the possibility of a leakage).

A table showing the energy consumption of the sensor node at different states, obtained from physical measurements, is provided in Table 4 below for analysis and for the validation of the energy consumption of the simulation model presented in Table 3.

The ESP32, when operating in modem sleep mode at a speed of either 80 MHz or 240 MHz, is not energy efficient and not suitable for a WSN application that is to be battery powered. However, as shown in the results in Figure 15, proper optimization and harnessing of the ULP coprocessor of the ESP32 while putting the ESP32 core to sleep can drastically reduce the power consumption, thus enabling it to be used for a battery-powered applications.

#### 6.5.3. Energy Budget Analysis and Validation of Simulation Model

In this sub-section, we perform an energy budget analysis of the sensor node obtained from physical measurements of the power consumption and also validate the simulation model of the sensor node’s power consumption.

Table 4 depicts the measured current consumption of the sensor node at different states when the ESP32 is operating at 80 MHz and 240 MHz clock speed.

As shown in Table 4, much of the sensor node’s energy is consumed when the radio is listening. The sensor node’s radio transceiver (nRF24L01+) has to always be in the listening mode so as to prevent the loss of packets since we are dealing with a real-time application and also because the communications are asynchronous. Even when we harness the ULP coprocessor of the ESP32 to reduce the node’s current consumption as shown in Figure 15, we still incur much current consumption from the radio transceiver which has to be left in listening mode (consuming 11.8 mA) in order to prevent the loss of packets. One way to reduce the power consumption of the sensor node can be to incorporate an ultra-low power wake-up receiver to the sensor node’s circuitry. By this, we can have the possibility of putting the nRF24L01+ transceiver in the power down mode (which consumes 900 nA), while still preventing the loss of packets required for our real-time application. The nRF24L01+ transceiver will only enter listening mode from power down mode when there is an interrupt from the wake-up receiver indicating the availability of a packet. Though this will make the sensor node more energy efficient and thus suitable for a battery-powered application, it will nevertheless increase the cost of the sensor node.

Furthermore, from the physical measurements taken when the ESP32 of the sensor node was operating at 80 MHz and 240 MHz, we observed that the node operating at 80 MHz is sufficient to run the DKF algorithm and consumes a current which is approximately half of what it consumes when operating at 240 MHz, as shown in Table 4.

Finally, when comparing the power consumption of the sensor node obtained from the model and those obtained from physical measurements, we realize that the power consumption of the node derived from the model is close to that obtained from physical experiments. For instance, the current consumption of the sensor node when transmitting is 91.1 mA derived from the model and 102 mA obtained from physical measurements. From these results, we realize that the power consumption of the sensor derived from simulation can provide us with a firsthand, yet precise, approximation of the power consumption of sensor nodes when embedded with every selected distributed algorithm we intend to study before physical implementation. This can be used to quickly evaluate the effect of every selected distributed algorithm’s power consumption on the lifespan of the WSN before physical implementation, thus revealing which distributed algorithm is more energy efficient. This model will be greatly utilized in a future study where we will evaluate a number of different DKF algorithms for their power consumption and their performance in reliably detecting leaks using low-cost vibration sensors.

### 6.6. Simulation of a Global Network

Now that we have demonstrated via simulations and physical experiments that the distributed data fusion capability of a DKF can be of interest in improving the reliability of leak detection using low-cost vibration sensors, and also established a model for the power consumption and validated it via physical experiments, we intend to extend our solution to a large-scale WSN. The goal is to measure interesting elements such as the lifetime of the network and also carry out real large-scale analyses.

**Figure 16:**
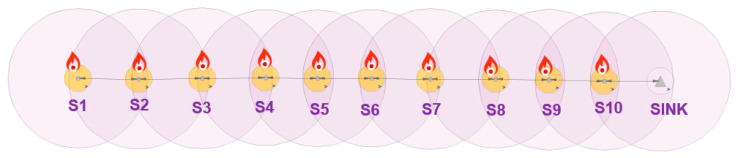
Simulation of a linear WSN consisting of ten sensor nodes and one sink node.

**Figure 17:**
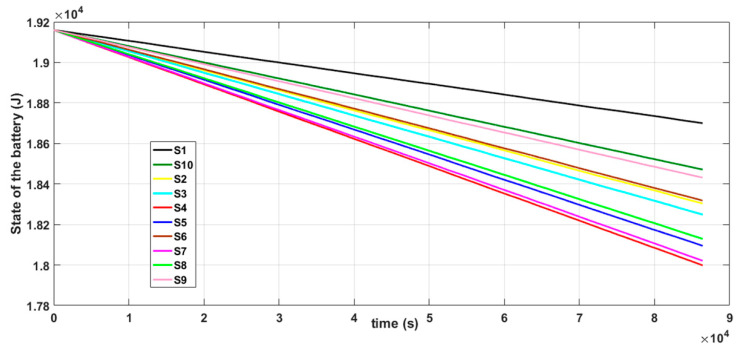
State of the battery of sensor nodes in distributed implementation.

**Figure 18:**
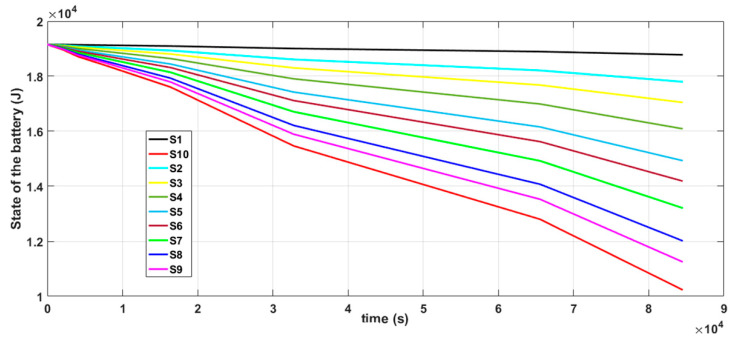
State of the battery of sensor nodes in centralized implementation.

In this section, we compare the power consumption and bandwidth consumption for the proposed distributed Kalman algorithm (that implements distributed data fusion) and the benchmark centralized Kalman filter algorithm (that implements centralized data fusion), in a sensor network consisting of 10 sensor nodes (S1–S10) connected in a linear topology and simulated in CupCarbon as shown in Figure 16. For the power consumption, we display the results of the state of the battery for each sensor node for both the cases where our proposed DKF algorithm and the benchmark centralized Kalman filter (CKF) were implemented. For the bandwidth consumption, we provide results for the number of messages transmitted for both the proposed DKF algorithm and the benchmark CKF. In the distributed implementation, the sink node is not involved, whereas in the centralized implementation, the sink node is involved in the fusion of the data obtained from all the sensor nodes. This requires numerous multi-hop communications and the sensor node (S10) directly connected to the sink node is involved in relaying the data from all the other sensor nodes to the sink. This makes S10 a critical node in the centralized implementation since it has a higher probability of developing an energy hole, which will affect the lifespan of the WSN. Figure 17 and Figure 18 display the simulation results of the energy profile for distributed and centralized solutions, respectively, for a simulation time of 1 day (86,400 s).

From Figure 17, we observe that, by implementing distributed computing using the proposed DKF algorithm, the rate of discharge of all the sensor nodes in the network is close to uniform. In addition, comparing the energy consumption of the proposed DKF algorithm (Figure 17) with that of CKF (Figure 18), we realize that the energy consumption of the proposed DKF is less than that of CKF. Comparing the results of Figure 17 and Figure 18, we realize at time *t* = 80,000 s, none of the sensor nodes in the DKF implementation have a battery state below 1.8 × 10^4^ J, whereas all the sensor nodes except S1 in CKF implementation had crossed that state at simulation time *t* = 80,000 s. S1 is an exception because it is involved in less multi-hop communications compared to the other nodes. From the results obtained, we observed that for a day period (86,400 s) sensor node S10 had exhausted 47.8% and 3.6% of its battery energy in the CKF and DKF implementations, respectively. We also observed that the nodes’ energy consumptions in the DKF are almost balanced and can lead to extension of the lifespan of the WSN since the likelihood of an energy hole developing is very low. However, in the CKF implementation, the nodes’ energy consumptions are not balanced. There is a greater likelihood of an energy hole occurring at S10. This shortens the lifespan of the WSN.

As a comparison of the bandwidth consumption of the DKF and CKF implementation, results in CupCarbon revealed that 198,380 and 3,265,479 messages were transmitted for the case of DKF and CKF implementations, respectively. From the results of the simulations, we observe that the bandwidth utilization of CKF is about 16 times that of the proposed DKF.

Though the CKF algorithm is Bayesian optimal, it has a very poor performance when it comes to energy consumption and bandwidth utilization. Other drawbacks are its lack of scalability, high degree of latency, and lack of robustness. This makes it an infeasible solution for large-scale WSNs.

### 6.7. Conclusion of Experimentation and Future Work

This study showed the feasibility of performing distributed computing in a WSN. With most of the state-of-the-art studies of distributed computing in WSN being theoretical and validated based on simulations, we performed the physical demonstration of distributed computing in WSN by implementing a DKF algorithm, proposed by Battistelli et al. in [58], to validate our simulation results. Simulations and physical experimentations were first performed on a two-node linear WSN and, finally, simulations and experimentation on a larger linear WSN are proposed for a future work.

The results obtained from simulations and field experiments reveal the importance of distributed data fusion in improving the reliability of the monitoring system when implementing distributed computing in WSN. Since the nodes make use of low-cost sensors and are deployed in environments where they can be exposed to circumstances that might interfere with measurements, the measurements may be imprecise at certain moments. In addition, even when environmental conditions are ideal, sensors may not give perfect measurements [38]. Data fusion can combine measurements from the multiple sources to obtain improved data that is of greater quality or greater relevance since all the sensors cannot be affected by noise to the same extent. Our results from both simulations and physical measurements prove this to be true. Furthermore, the sensing capability of each sensor node is restricted to a limited area. By fusing the data from a number of sensor nodes, each sensor node, to some extent, can have a global view of the overall parameter monitored and this can improve the reliability. However, the greater number of sensor nodes with incorrect data compared to the number of sensor nodes with correct data, the greater the reduction in the overall performance of the fusion process. This makes distributed data fusion, which limits fusion only among neighboring nodes, advantageous. A number of techniques for the distributed fusion of data from multiple low-cost sensor nodes exist. In [37], He et al. organized them into sequential-based, consensus-based, gossip-based and diffusion-based depending on how the nodes communicate with their neighbors in order to obtain optimal data from the fusion process. An optimal distributed data fusion algorithm will be one that that converges faster to the optimal value and requires less data transmission. In this study, the DKF algorithm proposed by Battistelli et al. [58], which we implemented, falls under diffusion-based distributed data fusion algorithms and distributed state estimation algorithms that limit the communication resources, according to the study of He et al. [37]. From the results we obtained, the implemented DKF algorithm [58] reliably separated a leakage scenario from a non-leakage scenario, it is fully distributed, which makes it scalable and useful for implementation in a fully distributed WWPM solution, and also has a low communication burden. It was selected for this initial investigation of how distributed data fusion affects the reliability of leak detection in WWPM because of its fully distributed nature and low communication burden resulting from its event-triggered communication ability. However, in a future study, we will experiment on other distributed data fusion algorithms with the best performances based on the results from [37] and investigate which of the distributed data fusion algorithms are most appropriate for our fully distributed solution for WWPM (i.e., the one that provides greater leak sensitivity, greater reliability in relation to leak detection, a lower response time and a lower power consumption).

## 7. Conclusions and Perspectives

In this study, we proposed a fully distributed solution for leak detection in WSN-based Water Pipeline Monitoring (WWPM). In our proposed solution, all the processing required for leak detection is performed at the sensor nodes, without needing a centralized station for the processing of leak signals. The aim was to eliminate multi-hop communications, reduce latency, reduce the sensor node’s power consumption and extend the lifespan of the WSN. We used a distributed Kalman filter (combination of a local filter and distributed data fusion) for processing the vibration signals read by an accelerometer attached to the pipe surface in order to detect the occurrence of leakages on the pipeline. Our results show the feasibility of applying distributed computing for leak detection in WWPM and establish the importance of distributed data fusion in improving the reliability of the leak detection system. In this study, we implemented a comprehensive design approach, involving simulations, system design, laboratory experimentation and test under real conditions. We also provided the power consumption of our solution, obtained via both simulations and physical measurements, and proposed ways to reduce the node’s power consumption in future work in order to extend the lifespan of the WSN.

In the future, we intend to improve the capacity of our laboratory testbed by deploying a larger linear WSN consisting of at least ten sensor nodes, where we will experiment on other DKF algorithms in order evaluate their performance and power consumption and finally define the best solutions for a fully distributed WWPM. The results of the best DKF will be compared with the results of other leak detection techniques already available in the literature in terms of sensitivity, accuracy, specificity and power consumption.

## Figures and Tables

**Table 1:** List of criteria for comparing selected WWPM.

Criterion	Description
Parameter monitored	This is a feature of the pipeline system that is detected by the sensor and used for leak detection after processing.
Sensor	Nature of the sensor used in the study to detect leak signals
Pipe material	Type of pipe that was used in the study. It can be metallic (e.g., steel) or plastic (e.g., Polyvinyl Chloride (PVC))
Pre-processing (PP)	Technique used for pre-processing (e.g., filtering) the leak signal
Leak Detection (LD)	Technique used for processing the leak signal to detect the presence or absence of a leak.
Leak Localization (LL)	Technique used for identifying the location of the leak.
Location of Processing	Processing can be done at the Base Station (BS), Fusion Center (FC) or at the Sensor Node (SN).
Monitoring type	Classifies pipeline monitoring into Centralized, Decentralized, or Distributed based on the location where processing takes place. Centralized: all processing takes place at the BS. Decentralized: part of the processing (PP and/or LD) take place at the SN and/or FC. Distributed: all processing takes place at the SN.

**Table 2:** Summary comparison of some selected studies in WWPM.

Ref	Monitored Parameter	Sensor	Pipe Material	Pre-Processing Technique	Leak Detection Algorithm	Leak Localization Algorithm	Location of Processing	Monitoring Type
[8]	Pipe’s surface vibration	Vibration Sensor (NEC Tokin)	N.A	Kalman filtering	Compression rates analysis	Graph-based technique	PP: SN LD: SN LL: BS	Decentralized
[25]	Pipe’s surface vibration	Accelerometer (MPU6050, ADXL335 and MMA7361)	Plastic (polyethylene)	N.A	Offline analysis	N.A	PP: N.A LD: BS LL: N.A	Centralized
[26]	Pressure	Force sensitive resistor	Plastic (polyethylene)	Kalman filtering and compression	Predictive Kalman Filter	Time of arrival difference	PP: SN LD: SN & FC LL: FC	Decentralized
[27]	Acoustic signals and pipe’s surface vibration	Hydrophones and accelerometers	Plastic (Polyvinyl Chloride)	Fast Fourier Transform (FFT) and compression	Acoustic leak detection technique	Cross-correlation	PP: SN LD: BS LL: BS	Decentralized
[28]	Pressure (Force sensitive resistor)	Temperature and pressure sensors	Plastic (Polyvinyl Chloride)	N.A	Relative pressure change	N.A	PP: N.A LD: BS LL: N.A	Centralized
[67]	Acoustic signals	Acoustic sensors	Metallic	N.A	Acoustic emission technique	Cross-correlation method	PP: N.A LD: BS LL: BS	Centralized
[68]	Pipe’s surface vibration	Accelerometer (KB12(VD))	Plastic (polyethylene)	Moving average	Fast Fourier Transform, Wavelet Transform, Power Spectral Density and Cross Spectral Density	N.A	PP: BS LD: BS LL: N.A	Centralized
[78]	Pipe’s surface vibration	Piezoelectric transducer	Plastic (Polyvinyl Chloride)	Amplification	Amplitude thresholding and FFT	Localization based on leak index	PP: BS LD: BS LL: BS	Centralized
[79]	Pipe’s surface vibration	IEPE accelerometer	Plastic (polyethylene)	Signal filtering and amplification	Standard deviation computation	N.A	PP: SN LD: BS LL: N.A	Centralized
[80]	Pipe’s surface vibration	vibration sensor	N.A	N.A	Power Spectral Density and Cross Spectral Density	Modified Maximum Likelihood prefilter	PP: N.A LD: BS LL: BS	Centralized

**Table 3:** Simulated current consumption of the sensor node at different states.

ESP32 Speed (MHz)	State	Current Consumption (mA)	Duration When Node Is at This State (msec)	Energy Consumption (mJ)
80	CPU idle + radio down	20	50	3.3
	CPU idle + radio listening	31.8	1000	105
	CPU active + radio listening	36.8	900	109
	CPU active + radio transmitting	48.1	50	7.9
240	CPU idle + radio down	40	50	6.6
	CPU idle + radio listening	51.8	1000	171
	CPU active + radio listening	79.8	900	237
	CPU active + radio transmitting	91.1	50	15

**Table 4:** Measured current consumption of the sensor node at different states.

ESP32 Speed (MHz)	State	Current Consumption (mA)	Duration When Node Is at This State (msec)	Energy Consumption (mJ)
80	CPU idle + radio down	23.7	N.A	N.A
	CPU idle + radio listening	31.8	1000	105
	CPU active + radio listening	35	900	104
	CPU active + radio transmitting	51	50	8.4
240	CPU idle + radio down	39	N.A	N.A
	CPU idle + radio listening	50	1000	165
	CPU active + radio listening	69.3	900	206
	CPU active + radio transmitting	102	50	16.8
